# Supplementary material for: Needle in a haystack: Harnessing AI in drug patent searches and prediction
Source: PLoS One. 2024 Dec 2;19(12):e0311238. doi: 10.1371/journal.pone.0311238 (PMC11611211; doi:10.1371/journal.pone.0311238)
Supplement: Attachment — Submitted filename: PONE-D-24-11668_reviewer.pdf [file pone.0311238.s001.pdf]

PLOS ONE

Needle in a haystack: Harnessing AI in drug patent searches and prediction

--Manuscript Draft--

**Manuscript Number:**	PONE-D-24-11668
**Article Type:**	Research Article
**Full Title:**	Needle in a haystack: Harnessing AI in drug patent searches and prediction
**Short Title:**	Needle in a haystack: harnessing AI
**Corresponding Author:**	Valbona Muzaka, Ph.D<br>Uppsala University: Uppsala Universitet<br>Uppsala, SWEDEN
**Keywords:**	natural language processing; artificial intelligence; chemical drug patents; pharmaceutical patents
**Abstract:**	The classification codes granted by patent offices are useful instruments for simplifying the bewildering variety of patents in existence. They are singularly unhelpful, however, in locating a specific subgroup of patents such as that of drug-related pharmaceutical patents for which no classification codes exist. The more these patents operate as valuable intangible assets, the more difficult it has become to identify and work with them. Taking advantage of advances in artificial intelligence and in natural language processing in particular, we offer a new method of identifying chemical drug-related patents in this article. The aim is primarily that of demonstrating how the proverbial needle in a haystack was identified, namely through leveraging the superb pattern-recognition abilities of the BERT (Bidirectional Encoder Representations from Transformers) algorithm, boosted in large part through fine-tuning them to recognise the patents in question. Achieving a relatively high accuracy score of 94; 40%, we are also keen to demonstrate some possible uses of the algorithm in the hope that both method and application will be further refined and expanded forthwith.
**Order of Authors:**	Leonardo Costa Ribeiro, Ph.D
	Valbona Muzaka, Ph.D
**Additional Information:**	
**Question**	**Response**
**Financial Disclosure**<br><br>Enter a financial disclosure statement that describes the sources of funding for the work included in this submission. Review the submission guidelines for detailed requirements. View published research articles from *PLOS ONE* for specific examples.<br><br>This statement is required for submission and **will appear in the published article** if the submission is accepted. Please make sure it is accurate.	Yes

**Funded studies**<br>Enter a statement with the following details:<br>• Initials of the authors who received each award<br>• Grant numbers awarded to each author<br>• The full name of each funder<br>• URL of each funder website<br>• Did the sponsors or funders play any role in the study design, data collection and analysis, decision to publish, or preparation of the manuscript?<br><br>Did you receive funding for this work?	
Please add funding details.<br>as follow-up to "**Financial Disclosure**<br><br>Enter a financial disclosure statement that describes the sources of funding for the work included in this submission. Review the submission guidelines for detailed requirements. View published research articles from *PLOS ONE* for specific examples.<br><br>This statement is required for submission and **will appear in the published article** if the submission is accepted. Please make sure it is accurate.<br><br>**Funded studies**<br>Enter a statement with the following details:<br>• Initials of the authors who received each award<br>• Grant numbers awarded to each author<br>• The full name of each funder<br>• URL of each funder website<br>• Did the sponsors or funders play any role in the study design, data collection and analysis, decision to publish, or preparation of the manuscript?<br><br>Did you receive funding for this work?"	Author who received the awards: LCR<br><br>The work undertaken for this paper was financed in part by the Coordenação de Aperfeiçoamento de Pessoal de Nível Superior (CAPES), grant 88887.837596/2023-00 and by Conselho Nacional de Desenvolvimento Científico e Tecnológico (CNPq), grant 312020/2021-0<br><br>The funders did not play any role in the study design, data collection and analysis, decision to publish, or preparation of the manuscript.<br><br>Funders websites:<br>CAPES: https://www.gov.br/capes/pt-br<br>CNPq: https://www.gov.br/cnpq/pt-br
Please select the country of your main research funder (please select carefully as in some cases this is used in fee calculation).	BRAZIL - BR

as follow-up to "**Financial Disclosure**<br><br>Enter a financial disclosure statement that describes the sources of funding for the work included in this submission. Review the submission guidelines for detailed requirements. View published research articles from *PLOS ONE* for specific examples.<br><br>This statement is required for submission and **will appear in the published article** if the submission is accepted. Please make sure it is accurate.<br><br>**Funded studies**<br>Enter a statement with the following details:<br>• Initials of the authors who received each award<br>• Grant numbers awarded to each author<br>• The full name of each funder<br>• URL of each funder website<br>• Did the sponsors or funders play any role in the study design, data collection and analysis, decision to publish, or preparation of the manuscript?<br><br>Did you receive funding for this work?"	
**Competing Interests**<br><br>Use the instructions below to enter a competing interest statement for this submission. On behalf of all authors, disclose any competing interests that could be perceived to bias this work—acknowledging all financial support and any other relevant financial or non-financial competing interests.<br><br>This statement is required for submission and **will appear in the published article** if the submission is accepted. Please make sure it is accurate and that any funding sources listed in your Funding Information later in the submission form are also declared in your Financial Disclosure statement.	The authors have declared that no competing interests exist.

View published research articles from [*PLOS ONE*]() for specific examples.<br><br>**NO authors have competing interests**<br><br>Enter: *The authors have declared that no competing interests exist.*<br><br>**Authors with competing interests**<br><br>Enter competing interest details beginning with this statement:<br><br>*I have read the journal's policy and the authors of this manuscript have the following competing interests: [insert competing interests here]*<br><br>* typeset	
**Ethics Statement**<br><br>Enter an ethics statement for this submission. This statement is required if the study involved:<br><br>• Human participants<br>• Human specimens or tissue<br>• Vertebrate animals or cephalopods<br>• Vertebrate embryos or tissues<br>• Field research<br><br>Write "N/A" if the submission does not require an ethics statement.<br><br>General guidance is provided below. Consult the submission guidelines for detailed instructions. **Make sure that all information entered here is included in the Methods section of the manuscript.**	N/A

Powered by Editorial Manager® and ProduXion Manager® from Aries Systems Corporation

**Format for specific study types**<br><br>**Human Subject Research (involving human participants and/or tissue)**<br>• Give the name of the institutional review board or ethics committee that approved the study<br>• Include the approval number and/or a statement indicating approval of this research<br>• Indicate the form of consent obtained (written/oral) or the reason that consent was not obtained (e.g. the data were analyzed anonymously)<br><br>**Animal Research (involving vertebrate animals, embryos or tissues)**<br>• Provide the name of the Institutional Animal Care and Use Committee (IACUC) or other relevant ethics board that reviewed the study protocol, and indicate whether they approved this research or granted a formal waiver of ethical approval<br>• Include an approval number if one was obtained<br>• If the study involved *non-human primates*, add *additional details* about animal welfare and steps taken to ameliorate suffering<br>• If anesthesia, euthanasia, or any kind of animal sacrifice is part of the study, include briefly which substances and/or methods were applied<br><br>**Field Research**<br><br>Include the following details if this study involves the collection of plant, animal, or other materials from a natural setting:<br>• Field permit number<br>• Name of the institution or relevant body that granted permission	
**Data Availability**<br><br>Authors are required to make all data underlying the findings described fully available, without restriction, and from the time of publication. PLOS allows rare exceptions to address legal and ethical concerns. See the PLOS Data Policy and FAQ for detailed information.	No - some restrictions will apply

Powered by Editorial Manager® and ProduXion Manager® from Aries Systems Corporation

A Data Availability Statement describing where the data can be found is required at submission. Your answers to this question constitute the Data Availability Statement and **will be published in the article**, if accepted.<br><br>**Important:** Stating 'data available on request from the author' is not sufficient. If your data are only available upon request, select 'No' for the first question and explain your exceptional situation in the text box.<br><br>Do the authors confirm that all data underlying the findings described in their manuscript are fully available without restriction?	
**Describe where the data may be found in full sentences. If you are copying our sample text, replace any instances of XXX with the appropriate details.**<br><br>• If the data are **held or will be held in a public repository**, include URLs, accession numbers or DOIs. If this information will only be available after acceptance, indicate this by ticking the box below. For example: *All XXX files are available from the XXX database (accession number(s) XXX, XXX.*).<br>• If the data are all contained **within the manuscript and/or Supporting Information files**, enter the following: *All relevant data are within the manuscript and its Supporting Information files.*<br>• If neither of these applies but you are able to provide **details of access elsewhere**, with or without limitations, please do so. For example:<br><br>*Data cannot be shared publicly because of [XXX]. Data are available from the XXX Institutional Data Access / Ethics Committee (contact via XXX) for researchers who meet the criteria for access to confidential data.*<br><br>*The data underlying the results presented in the study are available from (include the name of the third party*	We created a number of datasets to train our algorithm, as detailed in the article. Most of the data is publicly available, but some restrictions apply to other kinds of data, as explained below.<br><br>The Orange Book Database is publicly available and its URL is provided in the article.<br><br>The BERT algorithm is publicly available here: https://huggingface.co/docs/transformers/model_doc/bert<br><br>The Biomedical DistilBERT is also publicly available here: https://huggingface.co/nlpie/distil-biobert<br><br>To translate patent information from German to English we used Facebook's 667 state-of-the-art M2M-100 model, a non-English centric model which is available publicly at: https://huggingface.co/facebook/m2m100_418M<br><br>General patent information from USPTO is also publicly available and can be found at: https://ppubs.uspto.gov/pubwebapp/static/pages/landing.html<br><br>Patent information and related data pertaining to PATSTAT, DPMA (German Patent Office) and IQVIA is paywalled and not in the public domain. DPMA data was obtained from the PATSTAT database. This part of the data underlying the results presented in the study are available from these parties' websites, respectively at:<br><br>PATSTAT: https://www.epo.org/en/searching-for-patents/business/patstat<br><br>IQVIA: https://www.iqvia.com/

Powered by Editorial Manager® and ProduXion Manager® from Aries Systems Corporation

and contact information or URL).<br>• This text is appropriate if the data are owned by a third party and authors do not have permission to share the data.<br><br>* typeset	
Additional data availability information:	

Manuscript with authors details
Click here to access/download;Manuscript;Bert_Chemical_PLOS.pdf

Needle in a haystack: Harnessing AI in drug patent searches and prediction

Leonardo Costa Ribeiro[1], Valbona Muzaka[2*]

1 Departamento de Ciências Econômicas, Faculdade de Ciências Econômicas, Universidade Federal de Minas Gerais, Belo Horizonte, Minas Gerais, Brasil
2 Economic-history Department, Uppsala University, Uppsala, Sweden

◕The authors contributed equally to this work.
* valbona.muzaka@ekhist.uu.se

Abstract

The classification codes granted by patent offices are useful instruments for simplifying the bewildering variety of patents in existence. They are singularly unhelpful, however, in locating a specific subgroup of patents such as that of drug-related pharmaceutical patents for which no classification codes exist. The more these patents operate as valuable intangible assets, the more difficult it has become to identify and work with them. Taking advantage of advances in artificial intelligence and in natural language processing in particular, we offer a new method of identifying chemical drug-related patents in this article. The aim is primarily that of demonstrating how the proverbial needle in a haystack was identified, namely through leveraging the superb pattern-recognition abilities of the BERT (Bidirectional Encoder Representations from Transformers) algorithm, boosted in large part through fine-tuning them to recognise the patents in question. Achieving a relatively high accuracy score of $94,40\%$, we are also keen to demonstrate some possible uses of the algorithm in the hope that both method and application will be further refined and expanded forthwith.

Introduction

Well before the Covid-19 pandemic was declared, several entities filed patent applications related to the spike protein of the novel SARS-CoV-2 virus shortly after its publication in January 2020. Among these were companies such as Moderna and BioNTech, that would go on to gain market authorisation for their mRNA vaccines later that year, and scientists such as Katalin Karikó and Drew Weisman who won the Nobel Prize for their work on mRNA in 2023. The multiple legal challenges over mRNA patents that have followed since testify not only to the tension inherent in granting patent rights to specific assignees for cumulative and collaborative work developed over time – mRNA was discovered in 1960 and scientists have been working on developing mRNA vaccines with lipid-based delivery systems since the late 1970s [1] – but also to the recent transformation of pharmaceutical patents into highly valuable assets. Indeed, considerable amounts of public funding behind the knowledge embodied in the successful Covid-19 vaccines notwithstanding, it is the companies controlling the crucial patents and other forms of intellectual property (IP) that have reaped the Covid-19 vaccines' financial benefits.

Partly because of the socio-economic importance of the products the *proprietary* [1] pharmaceutical sector generates, and partly because of the important role patents have historically played in it, research on pharmaceutical patents is well-established in a number of disciplines, including philosophy, economics, business studies, law, and science and technology studies. However, scholars inclined to work with patents as their primary material – and users of the patent system at large – sooner or later encounter difficulties in identifying specific groups of pharmaceutical patents. One of the most of important patent groups, unsurprisingly for a sector focused on the development and introduction of new drugs, is that related to drugs.[2] Patent offices typically receive pharmaceutical patent applications related to a therapeutic agent well before it becomes embodied in a drug authorised for human use, and the attrition rate is high [2]. To be sure, such patents are categorised and indexed, but the problem persists for as we go on to explain in more detail, there is no specific patent classification for pharmaceutical drugs. Moreover, although the rights associated with a pharmaceutical patent are strictly national, the application process can follow national, regional and quasi-global routes (e.g. the International Patent Classification (IPC) system of the World Intellectual Property Organization, WIPO), thus resulting in a multiplicity of pharmaceutical patent codes, none of which is designated to pharmaceutical drugs. The distinct development trajectories of national patent laws and patent offices, the emergence of regional and international harmonisation initiatives, constant technological developments in the field, the rapid increase in pharmaceutical patent numbers worldwide, alongside insufficient attention to their indexing, have combined to make drug-related patents even harder to identify and therefore work with and analyse.

In the face of this mismatch between the importance of pharmaceutical drug patents – as highly valuable assets and as research objects – and their relatively poor searchability, this article proposes a method for facilitating their identification across the world. For reasons we come to below, the focus here is on small-molecule drugs derived from chemical synthesis (herein, chemical drugs); biologic drugs are usually large, complex molecules derived from living cells or through biological processes and pose a different set of challenges that we are currently working to address separately. The mainstay of the sector for over 100 years, chemical drugs still account for the majority of all drugs sold globally [3], despite the promise of biologics in time overtaking them in the pharmaceutical market. We find, however, that chemical drug patents constitute a relatively small share of pharmaceutical patents overall – a needle in a haystack - a somewhat surprising outcome for a sector heavily reliant on patents and focused on the introduction of new drugs.

We start by explaining the nature of the identified mismatch in the following section, before proposing artificial intelligence (AI) tools and natural language processing in particular as a way of rectifying it in the third one. The contribution this article makes is predominantly methodological, i.e. the introduction of a method for identifying a specific category of patents not captured by available patent classification codes. For this reason, we invest more time in explaining the BERT (Bidirectional Encoder Representations from Transformers) algorithm as a powerful natural language processing tool in section three, and the methodology we use to train it for the task at hand in section four. We obtain very promising results, namely a trained algorithm with

[1] The term is widely used in the literature to distinguish large, research-based, pharmaceutical companies reliant on proprietary intellectual property rights such as patents from other, most notably generic, companies.

[2] We use this term to include all medicines for *human* therapeutic use regardless of their type (e.g. chemical or biologic drug) and of their delivery method (e.g. pill, gel, injection etc.). A pharmaceutical drug patent would therefore include product, process, product-by-process, and method-of-use patents related to drugs approved for human use, broadly understood, but not diagnostic tools, instruments, plant or animal extracts, or what more generally can be understood as research tool patents that do not relate to a drug in particular.

an overall performance (F1) score of 0.94. Despite our focus on methodology, we are also keen to demonstrate some of its possible uses, which is why, after discussing our results in section five, we use the trained algorithm to identify chemical drug patents in two patent offices – the US and Germany ones – in the final section. Our method is inspired not by faith in AI tools like BERT possessing some form of superhuman cognition, but by their usefulness as knowledge instruments designed by us to address an identified problem. In explaining our model in detail we engage, inevitably, in a form of AI 'demystification', driven in part by a commitment to keep AI tools open.

Pharmaceutical drug patents in principle and practice

Couched predominantly in philosophical and legal terms, justifications for why patents are granted at all can be disarticulated from how patents operate in practice. Today, most pharmaceutical patents are used and managed as intangible assets, but the justification for their grant and enforcement through public law are the same as during the 19th century. As discussed in a seminal article by Machlup and Penrose [4], these were/are that: inventors have natural property rights over their inventive ideas; patent rights constitute a just reward to inventors; patents provide the necessary incentives for innovation; and, society grants time-limited monopoly rights to inventors in exchange for the latter making public their invention. The latter justification is central to the existence of public, searchable databases listing patents issued in all fields of technology, whereas the others are often merged in the utilitarian argument that (pharmaceutical) patents provide the necessary financial incentives – via higher profits – to develop new drugs that would otherwise not be developed due to increasingly complex and costly nature of pharmaceutical R&D.[3] Since Mansfield's classical study in 1986 [5], hardly anyone disagrees that the pharmaceutical sector is particularly reliant on patents due to a combination of relatively high costs for developing new drugs and their relatively high vulnerability to copying. Indeed, the sector stands alone in the extent of its involvement with the patent system and has done much to ensure that this system meets its requirements [6] [7].

A well-documented case of such involvement is the making of the 1994 WTO TRIPS (Trade-related Aspects of Intellectual Property Rights) Agreement which, as sector representatives were keen to boast, met most of the proprietary pharmaceutical sector's demands [8]. A key demand related to expanding the scope and geographical reach of all pharmaceutical patents led to a radical change in the global intellectual property (IP) regime. Before TRIPS made it obligatory on all its 164 signatories to offer pharmaceutical patents for 20 years, their duration varied greatly and was as low as five years in some countries, many offered only product patents, whereas others did not provide pharmaceutical patents at all [9]. Unsurprisingly, the number of *annual* pharmaceutical patent applications worldwide grew rapidly from under 20,000 in 1980 to around 100,000 in 2021.[4] At the same time, the link between the justification of patents on account of their innovation-stimulating effects and actual innovation appeared to have broken: the number of new drugs introduced annually in the market decreased from an average of 93 in the 1960s to 31 in the 1996-2014 period [10]. In the US, for instance, nearly 80% of the drugs associated with new patents in the 2000-2015 period were existing drugs in the FDA (Food and Drug Administration) records [11].

There are numerous explanations for the rapid increase in pharmaceutical patent

[3] For a recent articulation see, for instance, The IP Pact, issued by the largest proprietary pharmaceutical companies in 2021, available here https://www.interpat.org/ip-pact/

[4] Based on PATSTAT data, using IPC codes C07D, C07C, A61P and A61K.

numbers without the corresponding rise in R&D productivity. The practice of 'evergreening', extending the life of a drug patent by obtaining additional protections for minor modifications or additions, alongside that of 'patent fencing', obtaining many patent families covering various aspects of a single drug, are both well-known in the sector (e.g. [12] [13]), and help explain the multiplication of pharmaceutical patent numbers with no corresponding rise in R&D productivity. Humira, the autoimmune disorder drug that is one of the most profitable in history, illustrates well these strategies and its effects on competition and market dynamics: Abbott/AbbVie filed 247 patents – 90 % of which were filed after Humira was already in the market – and was granted 132 for various aspects of the drug, keeping Humira competitors off the market for a period likely to be 20 years beyond the primary patents term and guaranteeing enormous sale figures of US$133 billion in the 15 years since its introduction in 2003 [14]. Although biologic drugs tend to have more patents related to them, this is a problem affecting all drugs, especially the more successful ones. The twelve top-selling drugs in the US, for instance, held on average 71 patents per drug in 2018 [15].

A number of accounts have instead focused on the knowledge-specific difficulties associated with biopharmaceutical R&D processes to explain the phenomenon of disappointing R&D productivity (e.g. [16] [17]). Most accounts, however, tend to neglect broader structural changes that have occurred in the sector over the last three decades or so. Importantly, the core activities of large proprietary companies have shifted away from in-house R&D processes towards primarily leveraging knowledge in and through networks where much of research, development, and production activities now take place [18]. This new way of operating is based on large companies' control over and management of intangible assets of all kinds, of which IPRs in general and patents in particular are a significant category [19] [20]. Pharmaceutical patents – conceptualised both as legal entitlements and as material manifestations of future earning streams – are now largely constructed, bundled, and managed as assets critical to rent extraction in the sector (e.g. [21] [22] [23] [18]. This shift towards the control and management of intangible assets is visible in large companies' balance sheets: the ratio of intangible over total assets of the largest 27 proprietary pharmaceutical companies (controlling over 2/3 of the global pharmaceutical market) increased from an average of 13 percent in 2000 to 51 percent in 2018 [24]. This new way of operating remains very profitable for the proprietary pharmaceutical companies despite them introducing relatively fewer new drugs in the market: if anything, their profitability, historically above the average non-financial corporate sector, has increased even further in the recent decades [25]. This is line with evidence that the most profitable corporations currently are those that own or control IPRs and intangible assets [26] [27].

The disarticulation between patents' *raison d'être* and their current instantiation is at the heart of the mismatch we aim to rectify: the more pharmaceutical patents are managed as highly valuable assets, the higher the need of groups who work with them – researchers of various kinds, competitors, the legal profession, lawmakers, policymakers, civil society groups, society at large – to easily identify them. No difficulties exist in identifying pharmaceutical patents in general; using the main pharmaceutical patent codes, e.g. A61P, A61K, C07K, and C12N,[5] in any of the searchable patent databases provides a wealth of information on their number, main assignees, owners etc. But the classification categories do not enable the identification of specific pharmaceutical patent groups such as that of drug-related patents. A patent application related to a therapeutic agent is typically made before clinical trials for a treatment begin, and

[5]Common pharmaceutical sector codes in the IPC patent classification system are *A61P* (Medical or Veterinary Science: Specific Therapeutic Activity of Chemical Compounds or Medicinal Preparations), *A61K* (Medical or Veterinary Science: Preparations for Medical, Dental or Toiletry Purposes), *C07K* (Organic Chemistry: Peptides), and *C12N* (Microorganisms or Enzymes; Compositions Thereof; Propagating, Preserving, or Maintaining Microorganisms; Mutation or Genetic Engineering).

many such trials do not result in a drug approved for human use by the relevant authorities. A preliminary patent classification is first provided by the applicant and the relevant patent office then assigns classification numbers on the basis of patent legal claims so as to ease examiners' prior art search. More generally, classification codes granted by patent offices, despite their continues updates in response to technological changes, represent 'ideal' types that succeed in simplifying the bewildering variety of patents in existence. This simplification is useful for some purposes, but problematic for others, such as identifying the specific category of drug-related patents.

Precise classification is not merely a research problem. The pharmaceutical drug patent specifications are of crucial importance both at application/grant stage and at the enforcement stage; indeed, of the four patent justifications noted at the start, it is that of public disclosure that became over time the main one courts, especially in the Anglo-American tradition, came to rely upon [28] [29]. The legal literature on disclosure, for its part, laments that this important function of the patent system is limited in practice. Among other things, this is due to: the gaming of disclosure requirements to withhold commercially-valuable information; prioritising legal disclosure (patent claims) over technical disclosure so that the information contained is of limited practical use; the increasing complexity of legal patent claims aimed simultaneously at passing the originality test and expanding the outer limits of protected matter but without diminishing their defensibility in courts; and, importantly for our purposes, the difficulty and expense of locating patents in the vast database of issued patents (e.g. [28] [30] [29] [31] [15]). Not only is there insufficient attention paid to indexing patents and enhancing their searchability on the part of patent offices around the world; often enough, their classification can be arbitrary and therefore unreliable [32] [30]. In short, the opacity of patent texts, combined with their ever-increasing numbers and poor searchability, make patents uncertain entities for the majority of patent system users [33] [34] [29] precisely at a time when they have become crucial assets in the hands of large corporations.

There are four main reasons why identifying a sub-category of pharmaceutical drugs, such as drug-related ones, is worthwhile. First, developing new drugs is the core activity of the proprietary (as opposed to the generic) pharmaceutical sector. It is reasonable to assume that the majority of pharmaceutical patents would be drug-related, but our analysis indicates that they constitute a minority of pharmaceutical patents in general. Given that large proprietary pharmaceutical companies are the main ones that are able to introduce new drugs in the market, and that their high valuation and profitability relies to a considerable extent on IPRs in general and patents in particular, this raises the question of what non-drug related patents are issued for and how they are used in the sector. Second, and related, a strong justification offered for pharmaceutical patents, both by the state and the sector, is that they are needed to undertake ever more complex and expensive R&D that results in new and more effective drugs in the market. The basis of this position in the face of historically lower new-to-the market drug introduction rates and a majority of non-drug-related patents would require, minimally, a re-evaluation. Third, the question of which category of pharmaceutical patents are the most valuable cannot be answered without being able to clearly identify different subcategories. As recent mRNA patent litigation has shown, due to a combination of high value, high technical complexity, and relatively high defensibility in courts, drug-related patents appear to be among the most hard-fought patent litigation cases [35] [15], highlighting again the need to identify specific subcategories of pharmaceutical patents. Fourth, many jurisdictions allow for pre- and/or post-patent grant challenges and quite a number have been successfully attempted not only by competitors and generic companies, but also by health-orientated civil society groups. The proper functioning of these kinds of provisions relies on fully-searchable,

fine-indexed patent databases or, lacking these, as we indeed do, a reliable method of overcoming the difficulties that arise from relatively opaque and poorly indexed pharmaceutical patent data. These are the main reasons why we harness AI tools to enable the identification of drug-related patents.

We turn to AI and natural language processing (NLP) in particular not because we think it is a superhuman form of intelligence that can sort out the messy outcomes of historical contingencies in the field of pharmaceutical patents, but because it is a useful instrument that helps us secure better knowledge for our purposes. As we go on to show, NLP models and BERT in particular are very good at pattern recognition; their instrument-like nature is made clear in our account of BERT's training in which a considerable amount of 'craftmanship' is involved [36]. As with pharmaceutical patent databases, the way NLP models operate are often opaque, which is why we explain in detail how ours was built and trained. We start this process with the US patent office (USPTO); apart from the fact that the US constitutes the largest, most profitable, and hence the most attractive, pharmaceutical market in the world, the peculiar nature of the pharmaceutical patent application and drug approval processes there helps explain our chosen methodology, especially the training of the BERT algorithm.

Briefly put, the USPTO handles pharmaceutical patent applications and approvals and, as noted, they are submitted and granted years before there is a viable drug. Only a small part of this universe of drug-associated patents is of relevance when, following clinical trials, a new drug application is submitted for the purpose of gaining marketing approval by the US Food and Drug Administration (FDA). Under the 1984 Hatch-Waxman Act, new *chemical* drug sponsors, often large proprietary pharmaceutical companies, must list valid patents associated with the drug alongside their application to the FDA. Importantly, while the new drug sponsor has an obligation to provide and maintain accurate and complete drug patent information – e.g. patent number and its submission and expiry date for each of the patents related to specific aspects of the drug in the application – with the FDA, the role of the latter is purely ministerial, i.e. it is the responsibility of the new drug sponsor to determine and list relevant patents and their scope in the FDA application [15]. Should the new chemical drug be approved, the FDA merely publishes the list of patents related to the new drug as provided by the sponsor in the Approved Drug Products with Therapeutic Equivalence Evaluations, commonly known as the Orange Book. [6] The existence of the Orange Book is crucial to training our algorithm, since it is chemical drug-related patents we want to be able to identify. The equivalent for biologic drugs, the so-called Purple Book, [7] did not include such requirements and listing until 2020, this being a key reason why identifying biologic drug patents is relatively more difficult and why we address this challenge separately.

Methodology (I): Natural language processing

The most common way of searching patent databases – using keywords in the available searchable fields, namely the patent title and abstract – is also among the most ineffective in locating and identifying chemical drug patents. Even carefully selected keywords would miss many relevant patent documents. Keyword searches often do not allow for stemming – e.g. entering 'running' may not return documents containing 'run' or 'runner' – thus excluding potentially relevant findings from the list. More importantly, words often have multiple meanings that get determined by the

[6] US FDA Orange Book: Approved Drug Products with Therapeutic Equivalence Evaluations, https://www.accessdata.fda.gov/scripts/cder/ob/index.cfm, accessed on 13 November 2023.

[7] US FDA Purple Book: Database of Licensed Biologic Products, https://purplebooksearch.fda.gov/, accessed on 13 November 2023.

surrounding context – take the example of 'bank', an institution, a riverbank, a storage location – thus leading to the opposite problem of including many irrelevant results. Difficulties related to language are compounded by technological change. Even if the most effective keywords have been identified, technological change may have rendered them ineffective search tools, a problem especially pronounced in emerging technological fields, such as artificial intelligence or blockchain technology.

Our aim is to provide a more sophisticated method of identifying all patents related to chemical drugs through the use of the most advanced natural language processing tools currently available. Although not addressing this question, the only related study we are aware of is a short feature by Chien and colleagues [37] who, responding to a request by USPTO for input on improving the quality of drug patents before they are granted, offer a predictive model for determining which pharmaceutical patent will likely become drug patents using machine learning based on numerical (i.e. not textual) data. Its methodology is based on computing and analysing statistics for various aspects of patent documents, e.g. families, citations, parties involved, and ownership. Unlike them, we rely on the pre-trained language model BERT and fine-tune it for the task in hand, i.e. we re-train it to enable the classification of patents drawn from large, general patent databases as belonging to the group of chemical drug patents. The relevance and applications of this method, as already noted and as we go on to explain in more detail in the last section, are much broader than the specific task set out by the USPTO.

Before explaining the steps taken to train the algorithm for these purposes, we justify in this section our choice of BERT through a brief account of its superiority over other language processing tools. A good place to start is Word Embedding, a technique used in natural language processing (NLP) to represent words as numerical vectors in a high-dimensional space so that similar words are mapped to nearby points in it. The purpose is to capture the meaning and context of words in a way that can be understood by machines, enabling them to carry out various NLP tasks such as text classification, sentiment analysis, machine translation, and more. It addresses the problem afflicting more traditional techniques in which words would be represented as one-hot encodings that typically hinder machines' understanding of the text's meaning. Word embedding maps words to dense vectors in a high-dimensional space such that semantically similar words are clustered together. For example, words that have similar meanings, synonyms, antonyms, or words that appear in similar contexts, are mapped to nearby points in the vector space. This allows machines to capture the nuances of language and understand the relationships between words in a more human-like way.

Generally speaking, word embedding is a powerful technique for representing words as vectors, enabling machines to understand and process natural language more effectively. There are several techniques to learn word embeddings, including: (1) Word2Vec, a popular method which uses two different techniques, namely continuous bag of words (CBOW) to predict a target word based on its surrounding context, and skip-gram to predict the surrounding context based on a target word; (2) Global Vectors for Word Representation (GloVe) which represents words as vectors in a high-dimensional space, based on their co-occurrence patterns in a corpus of text; and, (3) FastText, a method for learning word embeddings from raw text data, which uses a hierarchical approach to represent words at multiple scales. The performance of NLP tasks is greatly improved through word embedding. It can handle out-of-vocabulary words better than traditional methods through representing unseen words as vectors that are close to seen words with similar meanings. In short, it is generally better at capturing the relationship between words, subtle differences, and language nuances compared to traditional methods.

Despite these strengths, however, word embedding techniques are not as sensitive to the context in which a word appears as we would like. Due to its focus on the

co-occurrence of words in a large corpus, word embedding is not as successful in capturing the different meanings of a word as used in a particular context. Going back to our earlier example of ‘bank’, its meaning would depend on the context in which it appears: ‘I swam across the river to reach the bank’ and ‘I drove across the river to reach the bank’ evidently refer to the riverbank and the financial institution respectively, with the verb determining the meaning in each instance. It is to address this lack of attention to context that attention-based models have been proposed. These models, often called Transformers, use attention mechanisms to focus on specific parts of the input text that are relevant and used them to transform the vector encoding of the other words in the sentence. These models enable some words (e.g. ‘drove’, ‘swam’) to alter the vector representation of others. Unlike recurrent neural networks, transformers are a type of neural network architecture that is particularly well-suited for sequence-to-sequence tasks, such as machine translation, summarisation, and text generation. This is because traditional recurrent neural networks process sequences one element at a time and use recurrent connections to capture long-term dependencies, whereas transformers process the entire input sequence in parallel and use self-attention mechanisms to weigh the importance of different parts of the input when generating the output.

Introduced initially by Vaswani et al. [38], transformers have become widely used in many NLP tasks. They consist of an encoder and a decoder, each composed of multiple identical layers. The encoder takes in a sequence of tokens (e.g., words embedding) and outputs a sequence of vectors, called ”keys,” ”values,” and ”queries.” The decoder then takes these vectors as input and generates an output sequence. The key innovation of transformers is the self-attention mechanism which, as noted, allows the model to attend to different parts of the input sequence simultaneously and generate output sequences of non-predefined length, capturing long-range dependencies and contextual information more effectively. Another important feature is their use of the positional encoding technique to preserve the order of the input sequence. Positional encoding adds a fixed vector to each input token, which encodes its position in the sequence. This allows the model to differentiate between tokens even if they have the same content. Because of these features, transformers have achieved state-of-the-art results on many NLP tasks, including machine translation, text generation, and question answering. Importantly for our purposes, they have also been used as building blocks for other models, such as RoBERTa (Robustly Optimized BERT Pretraining Approach) and BERT (Bidirectional Encoder Representations from Transformers), which have achieved even more remarkable results on a wide range of NLP tasks.

The BERT algorithm and its uses for text classification

Bidirectional Encoder Representations from Transformers, BERT for short, is a powerful NLP technique developed by Google in 2018. It is a pre-trained [8] language model that uses a multi-layer bidirectional transformer encoder to generate contextualised representations of words in a sentence. These representations can be fine-tuned for a wide range of NLP tasks, such as sentiment analysis, question-answering, language translation and, importantly for our purposes, text classification. A brief account of BERT’s architecture helps locate various features of the more advanced natural language processing techniques discussed so far, as well as

[8]Google pre-trained BERT using a corpus of text data known as the ‘Wikipedia + Books’ corpus. This corpus consists of approximately 45 million words in total drawn from around 4.5 million articles from Wikipedia articles harvested on August 9, 2017, and a 30,000-strong book collection in a plain text format drawn from the larger, free e-book database, Project Guthenberg. It was first pre-trained using a masked language modelling objective with the goal of predict the missing word in a sentence based on the context, although BERT is also trained to perform next sentence prediction tasks.

provide the necessary background for explaining how we fine-tune it for our purposes. Its architecture consists of the following main components:

- Input Embeddings: The input to BERT is a sequence of words represented as dense vectors (embeddings). These embeddings are usually learned using a Word2Vec or GloVe model mentioned earlier.
- Transformer Encoder: The input embeddings are fed into a multi-layer transformer encoder. Each layer in the encoder consists of two sub-layers: Self-Attention and Position-wise Feed-Forward Networks (FFN). As noted, the Self-Attention mechanism allows the model to weigh the importance of different words in the input sequence relative to each other and learn contextual relationships between them. The FFN processes the output of the Self-Attention layer to capture non-linear interactions between the input embeddings.
- Bidirectional Encoding: Unlike traditional language models that only consider the left or right context of a word, BERT uses a bidirectional approach that considers the entire input sequence in both forward and backward directions. This allows the model to capture both local and global dependencies between words.
- Pooler: The output of the transformer encoder is passed through a pooler layer, which aggregates the information from all positions in the sequence to form a fixed-length vector representation of the input text.

BERT has achieved a number of state-of-the-art results on numerous NLP benchmarks, including question answering (89,8% F1 score on the Stanford Question Answering Dataset [39]), named entity recognition (93.5% F1 score on the LINNEAUS dataset [40]), and text classification (98.9% accuracy on fake news detection [41]). But one of the most attractive features of BERT for our purposes is that it can be retrained to address new tasks while simultaneously leveraging the knowledge gained during the pre-training phase. Fine-tuning BERT to achieve more specific NLP tasks is achieved by adding an additional layer on top of the pooler layer to adapt the model to the specific downstream NLP task. This layer is called a Classifier. As we go on to show in the next section, the whole model needs to be retrained end-to-end using labelled data for the target task, in our case, classifying a pharmaceutical patent as a chemical drug patent.

To fine-tune BERT for text classification purposes, the classifier layer added on top of the pooler layer is a neural network with softmax activation function. This layer will associate the vector encoding obtained in the pooler layer to a label that represents the class to which we want to classify the texts, thus providing the probability of the (patent) text belonging to a certain class, in our case that of a chemical drug patent. Although to the best of our knowledge this is the first time BERT is fine-tuned for this purpose, interesting applications of BERT for text classification exist and include: (1) Spam vs. Non-Spam Emails (in one example, a team of researchers fine-tuned BERT on a dataset of 50,000 emails and achieved an F1 score of 0,969 [42]; (2) Medical Text Classification (such as identifying cancer types from clinical notes, outperforming other machine learning models in classifying breast cancer subtypes [43]; (3) Hate Speech Detection (in social media posts like Twitter, with a team achieving an F1 score of 0.91 through a fine-tuned BERT model [44]).

Methodology (II): Training the algorithm

Creating the training sets

To fine-tune the BERT algorithm for specific classification purposes, as discussed, the addition of a classifier layer implemented via a neural network is needed for which, in

turn, one or more training datasets are required. Ultimately with BERT, as with other NLP models, it is the quality of the training database that determines its accuracy. To train the algorithm to correctly classify chemical drug patents from the universe of pharmaceutical patents, building a pre-classified database containing chemical drug patents is a first crucial step. In the first instance, we turn to the FDA Orange Book discussed in the second section. We obtained its December 2022 version comprising approximately 5,000 patents related to approved chemical drugs. The training set is by no means complete, however, because the Orange Book list the relevant chemical drug patents but it lacks metadata. To obtain these patents' metadata, we turn to PATSTAT, a patent statistics database provided by the European Patent Office (EPO) containing data on patent applications and grants from various countries, including those filed under the WIPO Patent Cooperation Treaty. It contains data such as patent titles, inventors, applicants, assignees, priority patents, filing dates, grant dates, and citations for more than 100 million patents from more than 90 countries, including major patent-granting authorities such as the USPTO, the Japanese Patent Office, and the Chinese National Intellectual Property Administration. We use the patent numbers of the nearly 5,000 chemical drug patents listed in the Orange Book to extract their title and abstracts and additional metadata from PATSTAT, thus creating the first database of known chemical drug patents. This part of the training dataset is illustrated as the step 1 in Diagram 1. We opt to use patent abstracts to train the algorithm for two main reasons: first, they contain the main patent information in a shorter, summary form that helps reduce computational costs; and, second, as patent texts are much more likely to display local patenting idiosyncrasies, the use of more standardised abstract texts enhances the algorithm's applicability in patent offices around the world.

To train the algorithm comprehensively, a second database of a similar size is required. The first database includes documents we know are positively connected to chemical drugs and thus designated by the label 1. A second database would include pharmaceutical patents that lack explicit association with chemical drugs in the Orange Book, identified as 0 in our dataset. To compile this supplementary segment of the training data, we started with PATSTAT patents listed under pharmaceutical sector codes, but omitted the patents listed in the Orange Book thereby ensuring their affiliation with the pharmaceutical sector while excluding explicit association with chemical drugs. From this pool, we randomly selected approximately 5,000 patents, striking a balance between patents bearing labels 1 and 0 within our training dataset. This second part of the training dataset is illustrated as set 2 in Diagram 1. Through this process, the first training containing around 10,000 patents was built – referred to simply as the Orange Book Patents dataset below – but this is not the only training dataset we considered in our analysis. Driven by the need to train and evaluate the training dataset that would generate the best possible accuracy after training the BERT algorithm, we created two additional training datasets in order to compare the precision results of correctly identifying patents associated with chemical drugs obtained from each of them.

The second training dataset was also built from chemical drug patents listed in the Orange Book, but in this case we supplemented the set with other patents from the relevant patent families obtained from PATSTAT. A patent family refers to a group of patents filed domestically and/or in different countries or regions that often share the same priority date and/or cover the same or similar technical content. There is no agreed definition of a patent family, but the most common types are equivalent, extended, and single-priority families [45]. Generally speaking, the patent family provides legal protection for an innovation across multiple jurisdictions, allowing the owner to prevent others from making, using, or selling the invention without permission in all of them. As patent families may contain the same patent applied for and granted

in different offices, we included in this dataset only those that presented different abstracts to the ones we already had in the first training dataset so as avoid duplication. Around 8,000 patents associated directly or through their families with chemical drugs were obtained in this manner, which we labelled 1. This is also represented by the step 1 in Diagram 1, but related to a different training dataset. As previously, we created a randomly selected dataset of an equal number of patents from the pharmaceutical sector that had not yet been labelled as 1. This part of the training dataset is illustrated as set 2 on the Diagram 1. Therefore, our second training dataset – referred to as the 'Orange Book Patents & their Families' below – ended up having around 16,000 patents.

Diagram 1 – Illustration of the training process

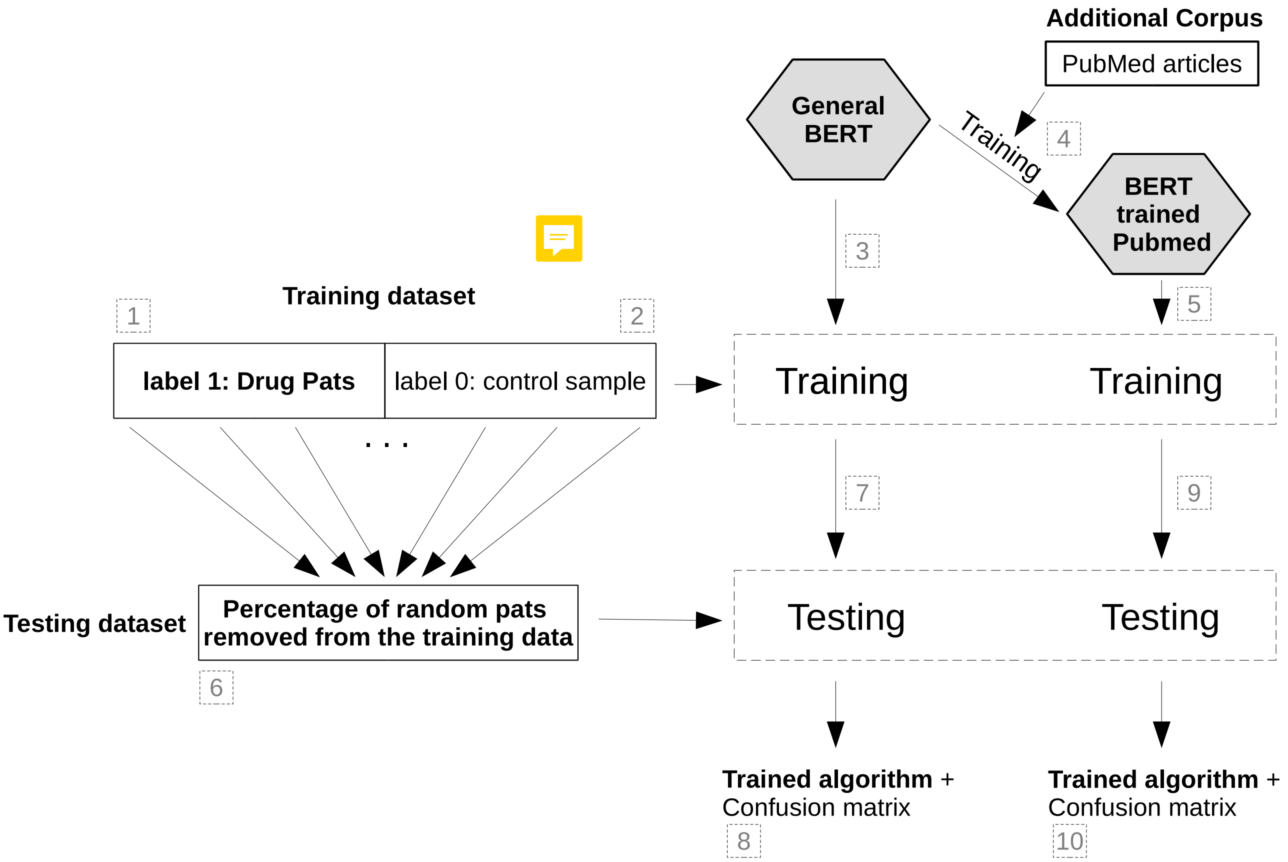

Source: Authors' elaboration

With an eye on improving accuracy, we built a third training dataset from a different source, namely IQVIA, [9] a database that tracks thousands of drug patent families across 130 countries. Using IQVIA own classifications which tend to include a larger number of patent family members, we identified just over 10,000 patents associated with major chemical drugs until 2019. We find that only 36% of these patents are present in our Orange Book database, making the use of the IQVIA database to train and improve the performance of our algorithm worthwhile. Using their patent numbers, we returned to PATSTAT to obtain the relevant metadata, including these patents' abstracts. This is also represented by the step 1 on the Diagram 1. As before, this training dataset was complemented by pairing this list of patents positively related to chemical drugs (labelled 1) with a set of around 10,000

[9] Provided by https://www.iqvia.com/, accessed on 15 Nov 2023.

patents randomly selected from PATSTAT that are classified as pharmaceutical sector patents but not yet identified as chemical drug patents (represented by the step 2 in Diagram 1). This third training dataset – corresponding to 'IQVIA Patents' in our discussion in section five – contained around 22,000 patents, i.e. it is over twice as large as the first 'Orange Book Patents' dataset discussed above.

Training the algorithm

The training of the algorithm using the datasets built earlier was conducted usi[illegible] Python programming language and the open-source implementation of DistilBE[illegible] provided by the HuggingFace library. DistilBERT is an optimised, streamlined variant of BERT, trained on the same comprehensive corpus as BERT discussed earlier, but requiring reduced computational requirements, therefore enabling the scale-up of the model with a feasible computation structure whilst offering the same precision of the original BERT algorithm. As noted earlier, one of the most attractive features of using BERT is that it can be retrained to address new tasks while simultaneously leveraging the knowledge gained during the pre-training phase. This ability to improve the performance of downstream tasks through leveraging previous knowledge is achieved through the so-called transfer learning mechanism, where the model uses the learned representations to adapt to new tasks with minimal additional training data. In short, the algorithm can be retrained with additional corpus, learning further specific semantic structures or meaning relations, adding these to what it had already learnt in previous training. This training stage is illustrated as the step 3 in Diagram 1.

It is this characteristic that Rohanian and colleagues [46] relied on to retrain the DistilBERT model using an additional corpus of articles published on PubMed. This retrained version [10] of DistilBERT – herein Biomedical DistilBERT – is particularly relevant for us because of the existing synergies between the corpus used to retrain it and our focus on chemical drug patents. PubMed has little to say on the latter but, boasting a vast collection of publications related to life sciences and biomedical subjects drawn from the MEDLINE database, it provides an excellent additional corpus to train this version on DistilBERT on the terms and language structures specific to the domain of biomedicine and life sciences. It is reasonable to expect this more fine-tuned version to be better capable of handling terms and syntactic structures specific to domains closely related to the subject matter contained in chemical drug patents. This additional training is illustrated as the step 4 in Diagram 1. What we are doing, in effect, is to increase the models' bias towards biomedical subjects with a view to improving its accuracy in identifying our pharmaceutical patent category. We test whether accuracy is improved by using both Biomedical DistilBERT and the general (i.e. non-PubMed-trained) DistilBERT version, the latter referred to as 'General DistilBERT' in our discussion in the following section. The step 5 in Diagram 1 corresponds to applying this fine-tuned version to our patent training dataset.

Results: assessing the identification performance

Aiming to train the BERT algorithm to correctly identify chemical drug patents from the universe of (pharmaceutical) patents, we built, as discussed, three different training patent datasets and used two versions of DistilBERT. These combinations enabled our AI model to be trained on a diverse range of patent information and enables us to evaluate their overall performance. To enable comparison between the different combinations/scenarios, we construct confusion matrixes for each of them, namely (1) Orange Book Patents dataset and General DistilBERT Corpus combination; (2) Orange

[10]Referenced as 'nlpie/bio-distilbert-uncased' in the HuggingFace library.

Book Patents dataset and Biomedical DistilBERT Corpus combination; (3) Orange Book Patents & their Families dataset and Biomedical DistilBERT Corpus combination; and (4) IQVIA Patents dataset and Biomedical DistilBERT Corpus one. All the results showed in the following section were obtained by running all combinations on a GPU with a batch size of 32, chosen due to memory limitations, and utilising 15 epochs due to the fact that the learning rate had already reached a significantly low value in all cases.

We use confusion matrixes because they offer clear, tabular representations measuring the performance of a classification model. Specifically, they show the count of true positives (TP, correctly predicted positive cases), true negatives (TN, correctly predicted negative cases), false positives (FN, incorrectly predicted positive cases), and false negatives (FN, incorrectly predicted negative cases). Given our objective of identifying chemical drug, the true positive (TP) corresponds to the cells in the lower right of the matrices presented in this section. The false positive (FP) corresponds to the upper-right cell, the false negative (FN) to the lower-left cell, and the true negative (TN) to the upper-left cells. Importantly for our purposes, confusion matrixes enable us to calculate several import metrics to access the performance of our proposed classification algorithm, namely:

- **Accuracy** – this measures the overall correctness of the model's predictions and is calculated as (TP+TN)/(TP+TN+FP+FN). It provides a general idea of how well the model is performing, but it may not be suitable when the class distribution is imbalanced.

- **Precision** – this measures the accuracy of positive predictions and is calculated as TP/(TP+FP). It indicates the proportion of positive predictions that were correct and is essential when minimising false positives is an important objective.

- **Recall/Sensitivity** – this measures the ability of the model to correctly identify all relevant positive instances and is calculated as TP/(TP+FN). It is crucial when avoiding missing positive instances is the main objective, even if it means including some false positives.

- **F1-Score** – this is the harmonic mean of Precision and Recall and is calculated as 2*(Precision*Recall)/(Precision+Recall). It is a particularly useful metric for evaluating overall performance that takes into account both false positives and false negatives. Because it provides a more comprehensive evaluation of the model's performance, we pay particular attention to the F1-score in the following analysis.

We start with the confusion matrix related to the scenario of using the Orange Book Patents training database discussed above and the General DistilBERT. Within the matrix, the integer values correspond to the number of patents correctly classified by the algorithm into eac[illegible]spective class, based on the testing dataset consisting of 1001 patents (10% of the tr[illegible]g dataset).[11] This testing dataset is illustrated as the step 6 in Diagram 1. The vertical labels indicate the true classifications of the patents, while the horizontal labels represent the algorithm's predicted classes. When calculating accuracy using the values presented in Figure 2 (represented by the steps 7 and 8 in Diagram 1), we obtain a result of 91.80%, i.e. this is the percentage of correct prediction rate for both positive and negative cases. The precision is 89.49%, representing the correct predictions within the positive class, and the recall is 93.66%, signifying the correct predictions within the category of actual medicine patents. Finally, the F1-score, which provides an overall performance assessment of the classification, is 0.915.

[11] The testing dataset is a collection of data used to evaluate the performance of a machine learning model. The purpose of a testing dataset is to assess how well the model can generalise to new, unseen data after it has been trained on a separate training dataset.

Fig 2. Confusion Matrix
Orange Book Patents – General DistilBert Corpus
Model Label T: OB; C: G

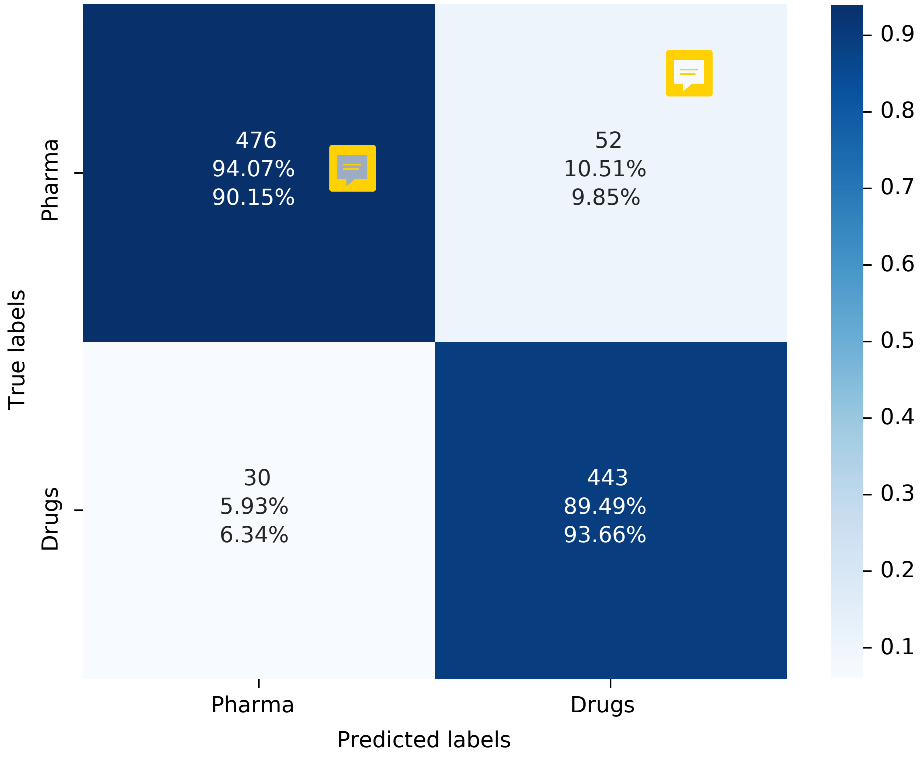

Source: Authors' elaboration

We build another confusion matrix, represented in Figure 3, for the scenario when the same training dataset as above was combined with Biomedical DistilBERT which, as discussed, was fine-tuned using the additional corpus of biomedical articles (represented by the steps 9 and 10 in Diagram 1). Now, when we calculate accuracy, we achieve a 93.40% result, along with 91.62% precision, 94.71% recall, and an F1-score of 0.931. Comparing these two scenarios, it is evident that all performance metrics improved with the use of the Biomedical DistilBERT version. In line with our expectations, the fact that this version of DistilBERT has been fine-tuned with a corpus of material that overlaps with the subject matter of the patents we want to classify, has contributed to improved performance metrics. This is why we do not use the General DistilBERT version in the remaining combinations.

The third combination involves the Orange Book Patents and their Families dataset and the Biomedical DistilBert Corpus. The confusion matrix for this model is displayed in Figure 4 (represented by the steps 9 and 10 in Diagram 1). To compute the confusion matrix, we utilised a testing dataset comprising 832 patents, which accounts for 0.5% of the total size of the initial training dataset. In this scenario, the accuracy is 91.34% result, the precision 85.88% precision, 92.26% recall, and an F1-score of 0.890. A comparison of the performance metrics of this model with the two preceding ones indicates that the inclusion of family abstracts in the training dataset had a negative impact on its performance. The probable cause of this phenomenon is the introduction of redundant information through the similarities shared among family abstracts which hinders the model's ability to generalize the behaviour of the training dataset to other

Fig 3. Confusion Matrix
Orange Book Patents – Biomedical DistilBert Corpus
Model label T: OB; C: G+B

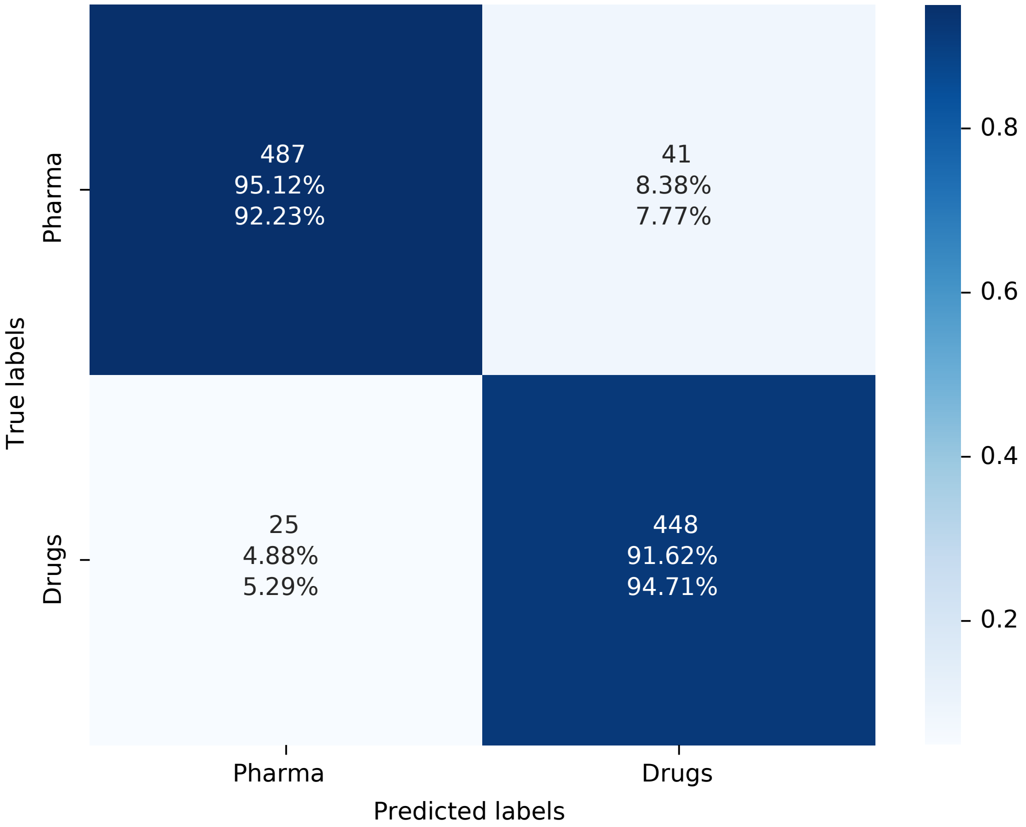

Source: Authors' elaboration

groups of patents (test dataset) effectively.

In our fourth and final combination, we used Biomedical DistilBERT because of its demonstrated higher efficiency in our previous results. But now we utilised the IQVIA chemical drug patent dataset as the training dataset which, as discussed earlier, it is twice as large as the Orange Book Patents' training dataset we used in the previous two models/scenarios (represented by the steps 9 and 10 in Diagram 1). Figure 5 illustrates the confusion matrix for this final model calculated on the testing dataset consisting of 1198 patents (5% of the training dataset). When calculating accuracy, we obtain a 94.40% result, 91.44% precision, 96.17% recall, and an F1-score of 0.937. Except for a minor decrease in precision, all other performance metrics showed improvement. This indicates that the increased volume of information in the latest training dataset has contributed to the better training of the algorithm, resulting in a subtle enhancement of its overall performance.

To enable meaningful comparisons between the performance metrics of various models, Table 1 displays all the relevant data. A notable enhancement in performance occurs when utilising the Biomedical DistilBERT Corpus. Conversely, there is a discernible decline in performance when incorporating the Orange Book patents and their families. The two most effective models deployed the Biomedical DistilBERT Corpus and either the Orange Book Patents or the IQVIA Patents databases for

Fig 4. Confusion Matrix
Orange Book Patents & their Families – Biomedical DistilBert Corpus
Model label T: OB+Family; C: G+B

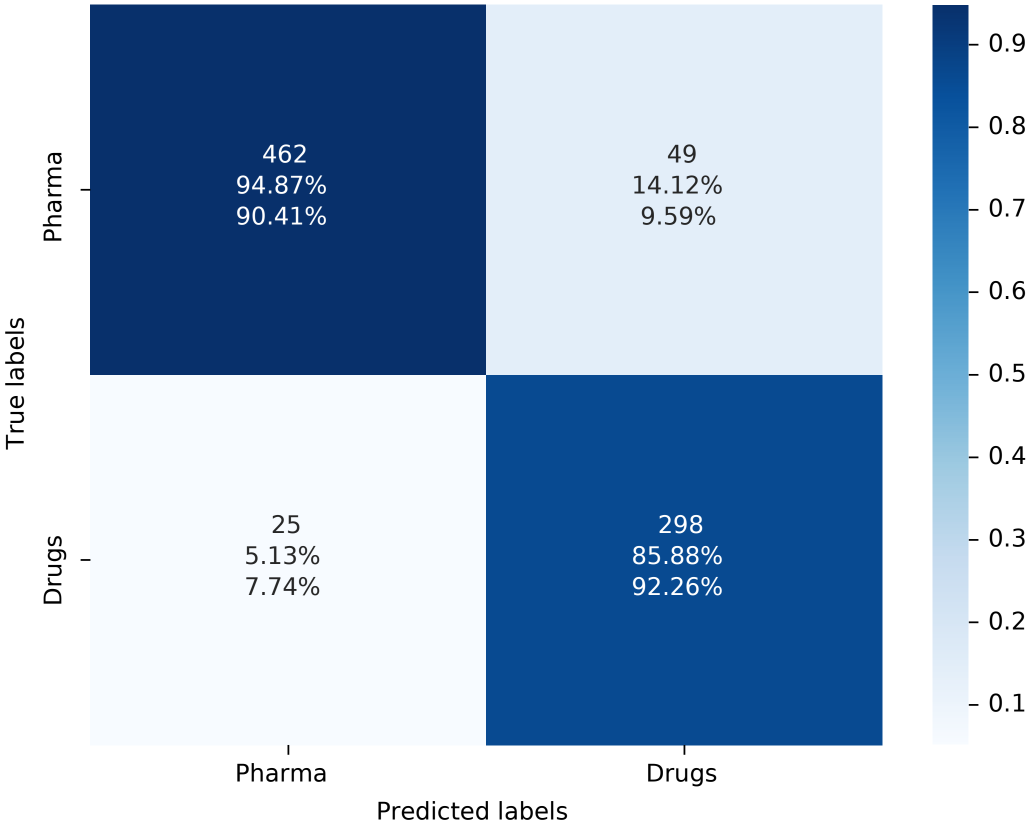

Source: Authors' elaboration

training. We opt for the latter combination because it exhibits a higher accuracy and recall compared to the former one. Moreover, its F1 score is superior, despite having a slightly lower precision score.

Table 1. Comparing the Models' Performance Metrics

Training Dataset	DistilBERT Corpus	Model label	Accuracy	Precision	Recall	F1-score
Orange Book Patents	General	T: OB; C: G	91.80%	89.49%	93.66%	0.915
Orange Book Patents	General + Biomedical	T: OB; C: G+B	93.40%	91.62%	94.71%	0.931
Orange Book & Family Patents	General + Biomedical	T: OB+Family; C: G+B	91.34%	85.88%	92.26%	0.89
IQVIA Patents	General + Biomedical	T: IQVIA; C: G+B	94.40%	91.44%	96.17%	0.937

Source: Authors' elaboration

Fig 5. Confusion Matrix
IQVIA Patents – Biomedical DistilBert Corpus
Model label T: IQVIA; C: G+B

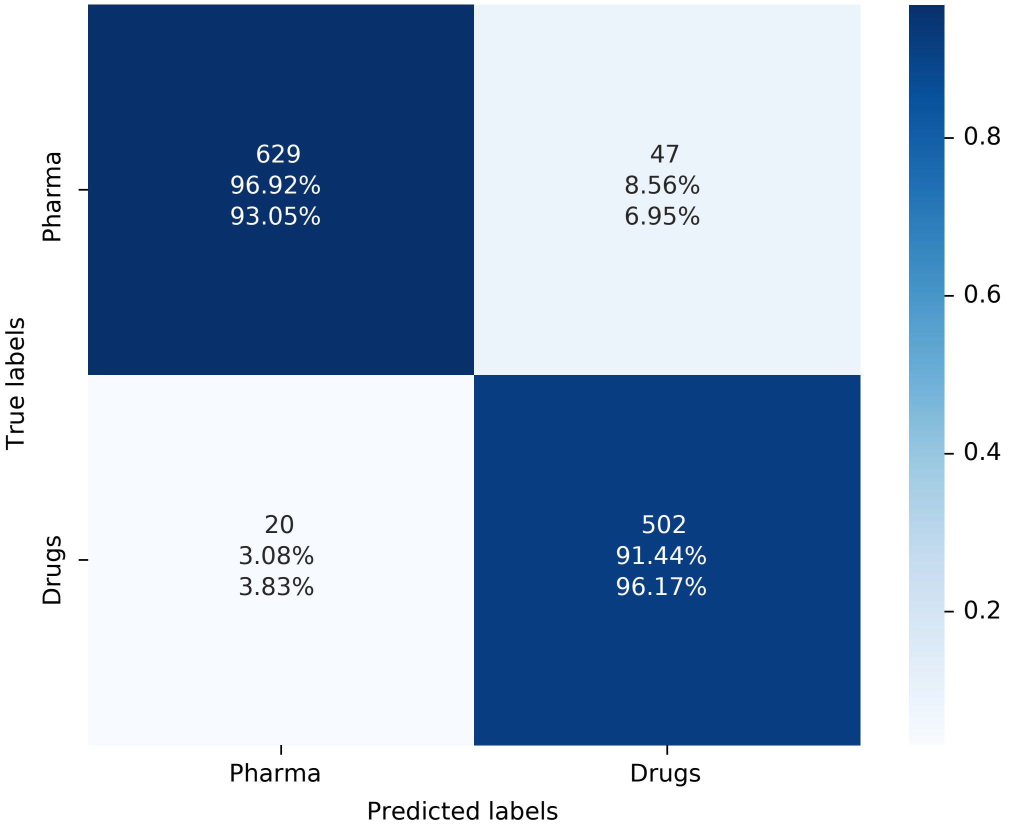

Source: Authors' elaboration

Applying the method to the USPTO and the German Patent Office

Various applications emerge once the algorithm has been trained to present high performance indices. It may seem counterintuitive to apply the algorithm to the USPTO in the first instance, as we do here, given that the Orange Book already lists granted chemical drug patents that correspond to FDA-approved drugs. There are, however, two reasons why this is appropriate. Firstly, there is the temporal gap between patent grant and drug approval which means that chemical drug patents listed in the Orange Book do not capture the entire universe of chemical drug-related patents in the US. As noted, a patent application related to a therapeutic agent comes early on in the process, typically before a decision to pursue the drug is made and clinical trials undertaken. Usually, only a very small proportion of promising compounds tested in-vitro and animals enters the clinical trials stage which typically last five to seven years [47]. Until the chemical drug is approved following successful clinical trials, many drug patents would appear in USPTO but not in the Orange Book. Leveraging our algorithm for recently-granted patents at the USPTO would thus capture almost all the universe of granted drug-related patents.

If we add to this temporal gap the time needed for the USPTO to grant the patent

in the first place – we calculate this based on USPTO data to be around three years – new potential uses emerge. Among the most intriguing results from applying our model to patents filed at the USPTO in the last decade, for instance, which would identify all the patent applications related to chemical drugs. Although we do not execute it here, longitudinal applications of this kind of proactive identification, combined with Orange Book data, can help identify the likelihood of a chemical drug-related patent application not only being granted but also being linked to an approved drug in the market at a future date, around ten[12] years later. This constitutes the second reason why we turn to the USPTO in the first instance. Research on the pharmaceutical R&D process suggests that only around 10-13% of compounds succeed in clinical trials [48] [47]; many candidates either prove unsuccessful or are discontinued by the drug sponsor for any reason, not least those related to potential financial returns. In short, if the success rate of clinical trials is correct, a considerable share of drug-related patents does not appear in the Orange Book at all.

Using our algorithm with the highest accuracy (labelled IQVIA Patents –Biomedical DistilBert Corpus, above) on a dataset of pharmaceutical patents[13] granted by the USPTO during 2010-2020, we identified 150,685 pharmace[illegible]al patents of which 14,348, or approximately 9.5%, are chemical drug-related p[illegible]nts. Some of them would be misclassified, but we know that their number is quite small due to the high-performance metrics detailed in the previous section. This percentage is surprisingly low[14] and, as noted, deserving of further investigation. Biologic drug-related patents would increase this share, especially as biologics tend to have more patents related to them, although not significantly given that there are relatively fewer biologic than chemical drugs in the market. These types of analyses are beyond the scope of this article; suffice to note that identifying the key patentholders within the drug-related subcategory, as we do in Table 2, would open fruitful avenues of research, including the temporal development of their drug-related patent portfolios.

Table 2. Top Drug Patentholders, USPTO 2010-2020

	**Firm Name**	**Country**	**Predicted Drug Patents**
1	NOVARTIS AG	CH	128
2	MERCK SHARP & DOHME	US	118
3	ALLERGAN INC	US	91
4	VERTEX PHARMA	US	89
5	BRISTOL MYERS SQUIBB CO	US	82
6	ABBVIE INC	US	74
7	GILEAD SCIENCES INC	US	73
8	ANTECIP BIOVENTURES II LLC	US	72
9	PURDUE PHARMA LP	US	70

Source: Authors' elaboration

Because our algorithm was trained on drug-related patent abstracts rather than metadata which is more likely to display local patenting characteristics, in principle it can be used in any patent office in the world. This application would allow the

[12]Three years needed, on average, for the USPTO to grant the drug-related patent, plus seven years, on average, needed to complete clinical trials and secure the FDA marketing approval.
[13]These are classified under IPC codes A61K, A61P, C07K, and C12N, as discussed in the second section
[14]The short article by Chien [37] claims that drug patents account for around 4% of total pharmaceutical patents. However, they do not explain how they reached this figure at all.

identification of drug-related patents in these offices and identify key patentholders and patenting strategies that potentially reflect more local or regional dynamics. We opted to use our algorithm to identify chemical drug-related patents in the German Patent Office (herein, DPMA), an important pharmaceutical locus in the EU. Two steps were undertaken first. To verify the applicability of our algorithm in patent offices besides the USPTO, we conducted an experiment using a random sample of 90 drug-related patent families, with each family having at least one patent granted by both the USPTO and the European patent Office (EPO). When the algorithm was applied to the abstracts of both the USPTO and EPO patents, it predicted the same classification for 93% of the families, supporting to our hypothesis that it can be effectively applied to patents from other patent offices. The second preparatory step relates to the language used by non-English language patent offices. We leveraged for this purpose Facebook's state-of-the-art M2M-100 model, a non-English centric model that can translate between 100 languages in any direction with high precision and accuracy.[15]

The DPMA lists a total of 5,750 pharmaceutical patent applications made between 2010 and 2020. Our algorithm identifies 395 of these as chemical drug-related patents, or approximately 6.9% of total pharmaceutical patent applications. Comparing the main applicants within this group of patents (Table 3) with that we obtain in the case of the USPTO (Table 2) brings to light a noticeable feature: while the USPTO list is dominated by high-profile, global proprietary pharmaceutical companies, the DPMA list includes companies with a seemingly more regional profile alongside prominent ones such as Bayer and Merck. Comparing top drug-related patent applicants identified by the algorithm to those of pharmaceutical patents leads to a number of interesting insights. First of these is that, both groups are more diverse in the case of the German patent office and not dominated by large proprietary pharmaceutical companies as in the case of the USPTO. The second is that companies that top the list of (general) pharmaceutical patents in the DPMA are not the same as those that top the drug-related patent lists. This suggests that, besides their more regional profile, there is also a specialisation occurring in terms of their predominant orientation. Third, and perhaps more important from a methodological point of view, is that the algorithm is identifying drug-related patents correctly in this case. This is visible in the fact that only around 1% of pharmaceutical patents of Henkel AG & Co, the company with the most patents, are identified as drug-related; conversely, this share is around 10% for Beiersdorf AG, the company with the largest number of drug-related drugs. In short, the algorithm is not simply attributing 6.9% of their patents as belonging to the drug-related patent category.

A final noteworthy feature of the results obtained from applying the algorithm to pharmaceutical patents at the DMPA is that knowledge protected by chemical drug-related patents in Germany is most likely to have developed domestically/regionally rather than elsewhere in the world and then protected in Germany via a priority application. This is because, when we examine the 395 chemical drug-related patents identified by the algorithm, we find that 348 of them (88%) do not have a priority claim, that is, they have been filed at the DMPA first and not associated with previously filed applications in other patent offices. This observation is in line with the more regional profile of the companies that top the list in the case of the DMPA compared to the USPTO. On the other hand, of the 14,348 drug-related patents the algorithm identified at the USPTO, $66,3\%$ list this office as their first priority. This lower percentage of a much larger body of drug-related patents held by more globally-oriented companies is in line with the US being the most important and

[15] Unlike traditional translation models that rely heavily on English-centric data, M2M-100 boasts a massive 7.5 billion sentence dataset spanning 100 languages, ensuring high accuracy across diverse linguistic domains.

Table 3. Top Chemical Drug-related and Top Pharma Applicants, DPMA 2010-2020

	Top Drug-related Patent Applicants		Predicted Drug Patents
1	BEIERSDORF AG	DE	57
2	BAYER SCHERING PHARMA AG	DE	31
3	HENKEL AG & CO KGAA	DE	20
4	FRESENIUS MEDICAL CARE DE GMBH	DE	8
5	ACROVIS BIOSTRUCTURES GMBH	DE	8
6	RATIOPHARM GMBH	DE	8
7	STADA ARZNEIMITTEL AG	DE	7
8	BAYER PHARMA AG	DE	6
9	LOHMANN THERAPIE SYST LTS	DE	6
10	MERCK PATENT GMBH	DE	6
11	AICURIS GMBH & CO KG	DE	6

	Top Pharma Patent Applicants	Pharma Patents
1	HENKEL AG & CO KGAA	1,808
2	BEIERSDORF AG	556
3	MERCK PATENT GMBH	89
4	EVONIK DEGUSSA GMBH	80
5	BAYER SCHERING PHARMA AG	77
6	FRAUNHOFER GES FORSCHUNG	72
7	BAYER PHARMA AG	70
8	BAYER IP GMBH	58
9	SIEMENS AG	58
10	SCHULZE ZUR WIESCHE ERIK	41
11	FORSCHUNGSZENTRUM JUELICH GMBH	38

Source: Authors' elaboration

dynamic pharmaceutical and patent market in the world.

Conclusion

We started with the observation of a mismatch between pharmaceutical patents as highly-valuable assets and their justification as a sort of compromise ultimately aimed at enabling new and more effective drugs reaching the market. Besides this, patent disclosure and knowledge diffusion remains an important, even if not the primary, feature of modern patent systems. Nevertheless, as noted, it is difficult to identify and work with specific categories of pharmaceutical patents beyond the rather crude ones given by patent offices. Focusing on chemical drug-related patents here, and on biologic drug-related patents in a separate forthcoming work, constitutes an attempt to make pharmaceutical patent data less opaque and closer to the disclosure and diffusion ideal of the patent system. Advances in AI, and in NLP in particular, offer us the opportunity to use these knowledge instruments more precisely to address the problem at hand. The ability to train the BERT algorithm to tackle particular tasks is especially useful. We leveraged this characteristic repeatedly by exposing it to additional texts containing terms and structures that are much more likely to be present in the patent subcategory in question, until we obtain the highest possible F1-score, combined with an accuracy of 94.40%. The peculiar nature of the drug R&D and patent grant processes mean that applying the algorithm to the USPTO database of pharmaceutical patents is a worthwhile endeavour. One interesting element that stems from the peculiar nature of these processes is that the algorithm can identify potential chemical drug-related patents up to ten years before drug approval, i.e. at the stage the patent application is made at the USPTO. Applying the algorithm to the German patent office revealed other features, the most relevant of which is perhaps the more regional nature of chemical drug R&D and patenting strategies of the companies involved. Throughout, we have been careful to make clear our 'craftmanship' and imprint on the algorithm's structure and training, and therefore on its outcomes. The detailed explanation of our

method constitutes an effort to keep AI tools open and to allow their scrutiny and evaluation by the broader community.

Supporting information

Acknowledgments

References

1. Dolgin E. The tangled history of mRNA vaccines. Nature. 2021;597:318–325.
2. Danzon P, Keuffel E. Regulation of the Pharmaceutical Industry. In: National Bureau on Economic Research Conference on Regulation. Cambridge, MA; 2005.
3. Makurvet FD. Biologics vs. small molecules: Drug costs and patient access. Medicine in Drug Discovery. 2021;9:100075.
4. Machlup F, Penrose E. The Patent Controversy in the Nineteenth Century. Journal of Economic History. 1950;10(1):1–29.
5. Mansfield E. Patents and Innovation: an empirical study. Management Science. 1986;32(2):173–181.
6. Macdonald S. Exploring the hidden costs of patents. 2002;.
7. Muzaka V. The Politics of Intellectual Property Rights and Access to Medicines. Palgrave Macmillan; 2011.
8. Sell S. Private Power, Public Law: the Globalization of Intellectual Property Rights. Cambridge: Cambridge University Press; 2003.
9. WIPO. Existence, Scope and Form of Generally Internationally Accepted and Applied Standards/Norms for the Protection of Intellectual Property; 1988. issued as GATT document MTN.GNG/NG11/W/24/Rev1.
10. Gassman O, Schuhmacher Aea. Leading Pharmaceutical Innovation: How to Win the Life Sciences Race. 3rd ed. Cham, SW: Springer; 2018.
11. Feldman R. May your drug price be evergreen. Journal of Law and the Biosciences. 2018; p. 590–647.
12. Faunce T, Lexchin J. "Linkage' pharmaceutical evergreening in Canada and Australia'. Australia and New Zealand Health Policy. 2007;4(8). doi:10.1186/1743-8462-4-8.
13. Hemphill CS. An Aggregate Approach to Antitrust: Using New Data and Rulemaking to Preserve Drug Competition. Columbia Law Review. 2009;109:629–688.
14. Richards K, Hickey K, Ward E. Drug Pricing and Pharmaceutical Patenting Practices. US Congressional Research Service; 2020. R46221.
15. Paradise J. Information Opacity in Biopharmaceutical: Innovation Through the Lens of COVID-19. American Journal of Law & Medicine. 2021;47:157–175.

16. Scannell JW, Blanckley A, Boldon H, Warrington B. Diagnosing the decline in pharmaceutical R&D efficiency. Nature Reviews Drug Discovery. 2012;11:191–201.

17. Scannell JW, Bosley J. Where Quality Beats Quantity: decision theory, drug discovery and the reproductibility crisis. PLoS One. 2016;11(2):1–21.

18. Muzaka V. The Knowledge-Leveraging Corporation in the Neoliberalisation Financialisation Nexus. Competition and Change. 2023;doi:10.1177/10245294231213414.

19. Schwartz H. Wealth and Secular Stagnation. The Russell Sage Foundation Journal of the Social Sciences. 2016;2(6):226–249.

20. Bryan D, Rafferty M, Wigan D. Intangible Capital. In: Seabrooke L, Wigan D, editors. Global Wealth Chains. Oxford: Oxford University Press; 2022.

21. Zeller C. From the gene to the globe: extracting rents based on intellectual property monopolies. Review of International Political Economy. 2008;15(1):86–115.

22. Coriat B, Weinstein O. Patent regimes, firms and the commodification of knowledge. Socio-Economic Review. 2012;10(2):267–292.

23. Roy V. A crisis for cures? In: Birch K, Muniesa F, editors. Assetisation: Turning Things into Assets. MIT Press; 2020.

24. Fernandez R, Klinge T. Private Gains We Can Ill Afford. 2020;.

25. Isik E, Orhangazi O. Profitability and Drug Discovery. Industrial and Corporate Change. 2022;31:891–904.

26. Manyika J, Ramaswamy S, Bughin J. Superstars: the dynamics of firms, sectors, and cities leading the global economy. McKinsey Global Institute Discussion Paper. 2018;.

27. Schwartz H. Global secular stagnation and the rise of intellectual property monopoly. Review of International Political Economy. 2022;29(5):1448–1476.

28. Roin BN. The Disclosure Function of the Patent System (or lack thereof). Harvard Law Review. 2005;118:2007–2028.

29. Drahos P. The Global Governance of Knowledge: patent offices and their clients. Cambridge: Cambridge University Press; 2010.

30. Fromer JC. Patent disclosure. Iowa Law Review. 2009;94(2):539–606.

31. Devlin A. The Misunderstood Function of Disclosure in Patent Law. Harvard Journal of Law & Technology. 2010;23(2):401–446.

32. Allison JR, Lemley M. The Growing Complexity of the United States Patent System. Boston University Law Review. 2002;82(1):77–144.

33. Jaffe A, Lerner J. Innovation and Its Discontents. Capitalism and Society. 2006;1(3):Article 3.

34. Thambisetty S. Patents as Credence Goods. Oxford Journal of Legal Studies. 2007;27:707–740.

35. Grabowski H, Brain C, Taub A, Guha R. Pharmaceutical Patent Challenges: Company Strategies and Litigation Outcomes. American Journal of Health Economics. 2017;3(1):33–59.

36. Pasquinelli M, Joler V. The Nooscope manifested: AI as instrument of knowledge extractivism. AI & Society. 2021;36:1263–1280.

37. Chien CHV, Halkowski N, Kuhn J. Distinguishing and Predicting Drug Patents. Nature Biotechnology. 2023;41:317–321.

38. Vaswani A, Shazeer N, Parmar N, Uszkoreit J, Jones L, Gomez AN, et al. Attention Is All You Need. arXiv preprint arXiv:170603762. 2017;.

39. Bhattacharjee S, Haque R, de Buy Wenniger GM, Way A. Investigating Query Expansion and Coreference Resolution in Question Answering on BERT. In: Métais E, Meziane F, Horacek H, Cimiano P, editors. Natural Language Processing and Information Systems. NLDB 2020. vol. 12089 of Lecture Notes in Computer Science. Springer; 2020.

40. Lee J, Yoon W, Kim S, Kim D, Kim S, So CH, et al. BioBERT: a pre-trained biomedical language representation model for biomedical text mining. Bioinformatics. 2020;36:1234–1240. doi:10.1093/bioinformatics/btz682.

41. Kaliyar RK, Goswami A, Narang P. FakeBERT: Fake news detection in social media with a BERT-based deep learning approach. Multimedia Tools and Applications;80.

42. AbdulNabi I, Yaseen Q. Spam Email Detection Using Deep Learning Techniques. Procedia Computer Science. 2021;184:853–858. doi:10.1016/j.procs.2021.03.107.

43. Zhang X, Zhang Y, Zhang Q, Ren Y, Qiu T, Ma J, et al. Extracting comprehensive clinical information for breast cancer using deep learning methods. International Journal of Medical Informatics. 2019;132.

44. Mozafari M, Farahbakhsh R, Crespi N. A BERT-Based Transfer Learning Approach for Hate Speech Detection in Online Social Media. In: Cherifi H, Gaito S, Mendes J, Moro E, Rocha L, editors. Complex Networks and Their Applications VIII. Springer; 2020.

45. Martinez C. Insight into Different Types of Patent Families. OECD Science, Technology and Industry Working Papers. 2010;(2010/02).

46. Rohanian O, Nouriborji M, Kouchaki S, Clifton DA. On the Effectiveness of Compact Biomedical Transformers; 2022. Available from: `https://arxiv.org/abs/2209.03182`.

47. Wong CH, Siah KW, Lo AW. Estimation of clinical trial success rates and related parameters. Biostatistics. 2019;20(2):273–286.

48. Hay M, Thomas DW, Craighead JL, Economides C, Rosenthal J. Clinical development success rates for investigational drugs. Nature Biotechnology. 2014;32:40–51.
