# Supplementary material for: Needle in a haystack: Harnessing AI in drug patent searches and prediction
Source: PLoS One. 2024 Dec 2;19(12):e0311238. doi: 10.1371/journal.pone.0311238 (PMC11611211; doi:10.1371/journal.pone.0311238)
Supplement: Attachment — Submitted filename: Response_to_Reviewers 260824.odt [file pone.0311238.s002.odt]

Response to the editors:
Thank you for the opportunity to think and improve further our arguments in the paper. We found the reviewers’ comments thoughtful and have responded to them in full, as detailed below. Our responses follow each comment as they appear in the PLOS ONE Decision email, reviewer #1 file PONE-D-24-11668_reviewer.pdf and in the reviewer’s comments. New sections and additions appear in blue in our resubmitted article and we also reproduce them here for your perusal. We are convinced the article is stronger as a result of these and other changes highlighted in the resubmitted version.

We look forward to your response,
L. Ribeiro and V. Muzaka


Journal requirements:
1. Please ensure that your manuscript meets PLOS ONE's style requirements, including those for file naming. The PLOS ONE style templates can be found at https://journals.plos.org/plosone/s/file?id=wjVg/PLOSOne_formatting_sample_main_body.pdf and https://journals.plos.org/plosone/s/file?id=ba62/PLOSOne_formatting_sample_title_authors_affiliations.pdf
Answer: We have adjusted the names of figures and the way they are cited to fit the PLOS One format. We have also made adjustments to the way the corresponding author's email is presented.
2. Please note that PLOS ONE has specific guidelines on code sharing for submissions in which author-generated code underpins the findings in the manuscript. In these cases, all author-generated code must be made available without restrictions upon publication of the work. Please review our guidelines at https://journals.plos.org/plosone/s/materials-and-software-sharing#loc-sharing-code and ensure that your code is shared in a way that follows best practice and facilitates reproducibility and reuse.
Answer: The authors are aware of the sharing of codes and will add them to the supplementary material if the article is accepted for publication.
3. We note that you have indicated that there are restrictions to data sharing for this study. PLOS only allows data to be available upon request if there are legal or ethical restrictions on sharing data publicly. For more information on unacceptable data access restrictions, please see http://journals.plos.org/plosone/s/data-availability#loc-unacceptable-data-access-restrictions.
Before we proceed with your manuscript, please address the following prompts:
a) If there are ethical or legal restrictions on sharing a de-identified data set, please explain them in detail (e.g., data contain potentially identifying or sensitive patient information, data are owned by a third-party organization, etc.) and who has imposed them (e.g., a Research Ethics Committee or Institutional Review Board, etc.). Please also provide contact information for a data access committee, ethics committee, or other institutional body to which data requests may be sent.
Answer: Of the three training datasets we used, one, and only one, was constructed from third-party data that cannot be shared publicly. That dataset is IQVIA/Ark. IQVIA is a private company that specialises in selling research focusing on the biopharmaceutical sector. Per IQVIA's licensing rules, data access and use are for the sole benefit of the client and cannot be shared with other parties. Due to these legal restrictions, we are unable to publicly share the third database, even if this would have been our preferred course of action. Should you with to contact the data provider to ascertain the veracity of these legal restrictions, please visit: https://www.iqvia.com/contact. While this is unfortunate, researchers who would like to reproduce the results of our research can use the first database in our study, which is publicly-available (see point b below), and which offers only slightly less favourable  performance metrics to the IQVIA one.
b) If there are no restrictions, please upload the minimal anonymized data set necessary to replicate your study findings to a stable, public repository and provide us with the relevant URLs, DOIs, or accession numbers. For a list of recommended repositories, please see
https://journals.plos.org/plosone/s/recommended-repositories. You also have the option of uploading the data as Supporting Information files, but we would recommend depositing data directly to a data repository if possible.
Answer: The other two training datasets were created using data that was publicly available from the EPO. Therefore, we have made the lists of patent publication numbers and their respective labels, which formed these training databases, available in the repository https://doi.org/10.7910/DVN/NNRNXJ
* Authors will make the repository available if and when the article is accepted for publication. 
4. PLOS requires an ORCID iD for the corresponding author in Editorial Manager on papers submitted after December 6th, 2016. Please ensure that you have an ORCID iD and that it is validated in Editorial Manager. To do this, go to ‘Update my Information’ (in the upper left-hand corner of the main menu), and click on the Fetch/Validate link next to the ORCID field. This will take you to the ORCID site and allow you to create a new iD or authenticate a pre-existing iD in Editorial Manager. Please see the following video for instructions on linking an ORCID iD to your Editorial Manager account: https://www.youtube.com/watch?v=_xcclfuvtxQ 
Answer: We added and validated the authors ORCID in the submission system.
5. Please amend either the abstract on the online submission form (via Edit Submission) or the abstract in the manuscript so that they are identical.
Answer: We copied the new abstract to the submission form.
6. Please review your reference list to ensure that it is complete and correct. If you have cited papers that have been retracted, please include the rationale for doing so in the manuscript text, or remove these references and replace them with relevant current references. Any changes to the reference list should be mentioned in the rebuttal letter that accompanies your revised manuscript. If you need to cite a retracted article, indicate the article’s retracted status in the References list and also include a citation and full reference for the retraction notice.
Answer: We checked and no retracted paper was cited. We removed some references following the suggestion to shorten the introductory section. The removed references are marked in the text with a strikethrough. As a standard practice for revised text, we have indicated new insertions in blue and deletions in red with a strikethrough.


Response to Reviewer #1:
Comment related to adjacent works: We thank this reviewer for the suggested readings. Upon consulting them, we added the following paragraph: “Ours is not the first attempt to use AI tools in patent data analysis. Lee and colleagues \cite{LEE2018}, for instance, used an artificial neural network to analyse USPTO patents in order to identify emerging technologies. They achieved this by using 18 numerical indicators calculated from patent metadata, e.g. number of citations, the Herfindahl index on classes of cited patents, number of inventors, number of claims, and so on. These were used to train a neural network and generate two quantitative indicators that measure a technology's 'emergingness' over time. Besides not focusing on pharmaceutical patents, they made no use of patent texts at all, unlike our proposed method. Another notable example is offered by Balsmeier and colleagues \cite{Balsmeier2018} who made use of a NLP model to visualise co-inventor networks and patenting trends in the US during the 1976-2016 period. This was achieved through using NLP to analyse the names of investors, patent holders and their locations, not too dissimilar a method from that used in the commercial AI tools for patent searches that have become available recently. Once again, no engagement occurs with the claims and content of the patents analysed, as our model does.” (pg. 6 of change-tracked file)
Line 25 (in file PONE-D-24-11668_reviewer.pdf): We removed the comma.
Line 44: We changed the semi-comma and lowercase b (; b) to dot uppercase b (. B).
Line 54: We change the first sentence of the paragraph to: "We start by explaining the nature of the identified mismatch in the following section, before proposing natural language processing, specifically, the BERT algorithm, as a way of rectifying it in the third one." (pg. 3 of change-tracked file)
Line 131: We agree with the annotation so no changes were made.
Line 258: We added the following clarification at the end of the paragraph: "Consider the field of artificial intelligence, for instance, where ten years ago keywords like "machine learning", "deep learning", and "natural language processing" may have been sufficient to retrieve the majority of the relevant patents. As the field evolved, however, new techniques and associated terminology have emerged, such as "Reinforcement Learning", "Generative Adversarial Networks," and "Transformers."" (pg. 7 of change-tracked file)
Line 261: We soften the statement by rewriting it as: "Our aim is to provide a more sophisticated method of identifying all patents related to chemical drugs by using recently developed and advanced natural language processing tools." (pg. 7 of change-tracked file)
Line 330: We added the following sentence to comment on the LSTM: "Furthermore, the attention mechanism is highly parallelizable compared to RNNs. It runs efficiently on a graphics processing unit (GPU) and does not face the vanishing gradient problem that the long short-term memory (LSTM) neural network does." (pg. 8 of change-tracked file)
Line 338: We added the reference [44], [45], [46] at this point. (line 364 pg. 8 of change-tracked file)
Line 353: We have rewritten the item to make it more precise and clearer as follows: "BERT creates its own word embedding (vector representation of the words as Word2Vec and Glove) that is more sophisticated than the ones mentioned earlier. It uses a combination of token embeddings, segment embeddings, and positional embeddings to represent each token in the input. For words that are out of vocabulary, BERT word embedding splits them into subword tokens." (pg. 9 of change-tracked file)
Line 369: We added the following text to emphasize that the classification dense layer is part of the pooler: "This component can also include a neural network dense layer that performs the final classification, i.e., associates the transformer encoder with the class labels." (pg. 10 of change-tracked file). We also change the word "added" to "inserted" in line 411 to emphasize it. Finally, we also added the interpretation of the [CLS] token in the answer related to the comment of line 608. See pg. 17 of change-tracked file.
Line 378: We changed Classifier to classifier. (line 412 of change tracked file)
Line 380: We added "i.e., a binary classification" at the end of the sentence to clarify that it is a binary classification. (line 415 of change-tracked file)
Line 430: We would like to clarify that all Orange Book patents were added to the training dataset; no ablation was implemented in this case. To improve reproducibility, we added the following text detailing the pharmaceutical patents' sample selection:  “This sample was obtained from a random selection using a uniform probability distribution among patents granted by the USPTO in the period from 1980 to 2023 (same temporal coverage as the Orange Book) and classified in IPC codes, C07K, C12N, A61K and A61O, the most commonly used codes in the pharmaceutical sector.” (pg. 12 of change tracked file)
Line 439: We use the term "transfer learning" to introduce the use of Biomedical DistilBERT. In this approach, Rohanian and colleagues modified the weights of the general DistilBERT model, which was originally trained with Wikipedia data and others, by fine-tuning the model with a specific corpus of scientific papers from medicine and biology.
Comment in Diagram 1: We created a new diagram improving the previous version to make it clearer. We also adapted the steps numeration on the text to correspond to the new diagram. (pg. 13 of change-tracked file)
Line 476: We added an explanation of the distillation processes in BERT to the methodological section. The new part is on page 10 of the change tracked version of the manuscript, and we transcribe it here: "Distillation is a method used to transfer knowledge from a large, complex model (the "teacher" model) to a smaller, simpler model (the "student" model) while maintaining performance. In the case of BERT, distillation is used to condense the knowledge of a large, pre-trained BERT model (the teacher) into a smaller, more efficient model (the student). This process involves training the student model to emulate the behaviour of the teacher model using fewer parameters and computations [45].
The distillation process for BERT entails using a large, pre-trained BERT model as the teacher model, which has been trained on extensive datasets, as described earlier. The student model, on the other hand, is designed to be simpler with fewer parameters and layers, making it faster and more efficient. During training, the student model aims to predict output probabilities similar to those of the teacher model by minimizing the difference between their output distributions. This is achieved through a loss function called the knowledge distillation loss, typically a combination of cross-entropy loss and Kullback-Leibler divergence. By minimizing this loss, the student model is trained to match the output distributions of the teacher model.
One popular example of BERT distillation is DistilBERT, which employs knowledge distillation during the pre-training phase to reduce the size of the BERT model by 40% while retaining 97% of its language understanding capabilities and achieving a 60% improvement in speed [45].
Once the student model is trained, it can be fine-tuned for specific tasks, such as sentiment analysis or question-answering, as the regular BERT algorithm."
Line 503: We re-wrote the sentence as "In essence, we are increasing the models' capability to handle biomedical subjects to improve its accuracy in identifying our pharmaceutical patent category." (pg. 14 of change-tracked file)
Line 555: We added the following footnote justifying the use of 90%-10% division between training and testing datasets: "Different percentages for dividing the training and testing sets were considered. The final decision was to use a 90%—10% separation because the model's accuracy plateaus above 90% of the training set, and the statistical fluctuation increases when the test set is relatively small." (pg. 15 of change-tracked file)

Comment on Figure 2:
Comment in 1st cell of Figure 2: We explain in each figure what each percentage refers to. The first value in each cell refers to the percentage in relation to the sum of the respective column. And the second, to the percentage in relation to the sum of the respective row.
Comment in 2nd cell of Figure 2: We added the following paragraph related to the misclassified abstract analysis: “Misclassified patents were found upon further inspection to contain quite generic texts, this probably explaining the misclassification. False positives can in part be explained by the fact that there will be patents that are related to new drugs that have not yet completed the FDA approval process. These patents, while relevant, are not yet included in the Orange Book database and hence are not labelled as drug patents in our training dataset.” (pg. 15 of change-tracked file)
Comment on Figure 3: We created more descriptive captions for all figures.
Line 608: We added the requested analysis, and the only difference was that we used UMAP as a space dimensionality reduction technique instead of T-SNE. To accomplish this, we extracted the embedding corresponding to the [CLS] token (the first token in the sequence) for each abstract in the training dataset from the model's pooler. The [CLS] embedding is often used as a representation of the entire sequence, serving as the vector representation of the whole abstract in this case. Subsequently, we applied UMAP to reduce the dimensionality of the space from 768 (the size of the BERT embeddings) to 2 dimensions. We then constructed a scatterplot in this reduced space, with pharmaceutical patent abstract embeddings represented in blue and drug patents in orange.
We added the following text: “To enable the visualization of the classification performed by the most efficient model (the last one) for recognizing the two different patent groups using BERT, we began by getting the representation of the embeddings in the BERT pooler layer before applying the last layer of a dense neural network for patent classification. We then projected the embeddings into a two-dimensional space using the Uniform Manifold Approximation and Projection (UMAP), also an AI algorithm. The UMAP algorithm is used to reduce high-dimensional data into a lower-dimensional space while retaining the essential structure and relationships within the data. UMAP accomplishes this by modelling the data's local relationships using nearest-neighbour graphs and optimizing a low-dimensional representation that maintains these local relationships as closely as possible. This is achieved through an iterative process of adjusting the positions of the data points in the lower-dimensional space in order to preserve similar relationships and distinguish dissimilar ones. This approach makes UMAP effective for visualization, clustering, and preserving the global and local structure of the data in a lower-dimensional space. For the projection, we extracted the embedding corresponding to the [CLS] token (the first token in the sequence) for each abstract in the training dataset from the model's pooler. The [CLS] embedding is often used as a representation of the entire sequence, serving as the vector representation of the whole abstract in this case. Subsequently, we applied UMAP to reduce the dimensionality of the space from 768 (the size of the BERT embeddings) to 2 dimensions.
Finally, we constructed a scatter-plot in this reduced space, with pharmaceutical patent abstract embeddings represented by the blue circles and drug patents by orange circles. This information we took from the real labels in the training base.
In Fig 6, this scatterplot clearly shows the separation between pharmaceutical and drug patents. Pharmaceutical patents are concentrated in the centre of the graph, while drug patents are spread around this central group but clearly separated from it. There are very few points associated with drug patents in the region where pharmaceutical patents are concentrated, and there are very few blue dots associated with medicine patents in the region. This indicates that the classification model has achieved high precision.” (pg. 17 of change tracked file)
Line 643: We had linked the percentage we found with Chien's in footnote 14. So we brought this discussion into the body of the text to draw more attention to it. We added the following text to the text body: “Chien claims that drug patents account for around 4% of total pharmaceutical patents; however, the method used to achieve this result was not explained, making direct comparisons with our finding difficult” (pg. 21 of change-tracked file)
Line 669: We soften the highlight on the high precision of M2M-100 model. See pg. 22 of change tracked file.


Response to Reviewer #2:
Comment 1. Abstract: The abstract is not cohesive, can be revised to overall summarize the outcome of the analyses.
Answer: we have revised the abstract to better reflect our findings. It now reads: “The classification codes granted by patent offices are useful instruments for simplifying the bewildering variety of patents in existence. They are singularly unhelpful, however, in locating a specific subgroup of patents such as that of drug-related pharmaceutical patents for which no classification codes exist. Taking advantage of advances in artificial intelligence and in natural language processing in particular, we offer a new method of identifying chemical drug-related patents in this article. The aim is primarily that of demonstrating how the proverbial needle in a haystack was identified, namely through leveraging the superb pattern-recognition abilities of the BERT (Bidirectional Encoder Representations from Transformers) algorithm. We build three different databases to train our algorithm and fine-tune its abilities to identify the patent group in question by exposing it to additional texts containing structures that are much more likely to be present in them, until we obtain the highest possible F1-score, combined with an accuracy of $94.40\%$.  We also demonstrate some possible uses of the algorithm. Its application to the US patent office database enables the identification of potential chemical drug patents up to ten years before drug approval,  whereas its application to the German patent office reveals the regional nature of drug R\&D and patenting strategies. The hope is that both the method proposed and its applications will be further refined and expanded forthwith.” (pg. 1 of change-tracked file)

Comment 2. Introduction: This section is unnecessarily long. For ex. The TRIPS section (page 3, last para) seems irrelevant and can be removed. The other paragraphs can also be squeezed.
Answer: We assume this comment refer to the first section as the introduction is relatively short and each paragraph is dedicated to laying out the problem at hand. E.g. the first one introduces pharmaceutical patents as valuable assets by placing them in the immediately recognizable context of the recent pandemic, the second their poor searchability, and the other two paragraphs explain the structure of the article and the contribution it seeks to make. We did, however, made the first section – Pharmaceutical drug patents in principle and practice – more succinct by removing some parts and rewriting others. We are pleased with the result: a section which is shorter but nevertheless explains in some detail the mismatch we identify at the start and promise to explain here. In essence, we want to show that pharmaceutical patents are justified in one set of ways, but are used in another in the sector, namely as valuable intangible assets. This mismatch is an important reason why we embark on our journey. Another reason for providing this account at all is because we want to article to be read and perused by a wide readership, i.e. not just those interested in AI tools and their use in patent data analysis, but also those working on pharmaceutical patents from other disciplines. Finally, this multidisciplinary way of constructing and looking at the problem is important to us as authors and reflects our research interest and profiles.  
Comment 3. Figure 1, pg 11: The figure legend needs to be self-explanatory so that readers get an idea of what has been done without reading the methods section. Also the text “Source: Authors’ elaboration can be removed. ”
Answer: We have constructed a new figure 1 with a more self-explanatory diagram. See the new Figure 1 on page 13 of change tracked file. We have also removed the “Source: Authors’ elaboration"
Comment 4. The manuscript is not as per PlosOne format. i.e. footnotes not encouraged, references in Vancouver style etc. This needs to be considered.
Answer: We have significantly reduced the number of footnotes by moving them into the body of the text. We built the manuscript using Latex and the template provided on the PLOS One website. This template uses a custom style for generating references called plos2015, also provided on the PLOS One website. We then switched from plos2015 to vancouver by commenting out the \bibliographystyle{plos2015} command in the journal template and adding the \bibliographystyle{vancouver} command.

Response to Reviewer #3:
Comment 1. There are currently a number of widely available AI tools for patent searching. A discussion around these would benefit this article by providing the advancement of the AI tool developed in this study compared to other available AI tools for patent searching.
Answer: This is similar to the concern raised by reviewer 1. We added the following text to address these AI tool for patent searching and highlight the difference among them and our proposal: “Ours is not the first attempt to use AI tools in patent data analysis. Lee and colleagues \cite{LEE2018}, for instance, used an artificial neural network to analyse USPTO patents in order to identify emerging technologies. They achieved this by using 18 numerical indicators calculated from patent metadata, e.g. number of citations, the Herfindahl index on classes of cited patents, number of inventors, number of claims, and so on. These were used to train a neural network and generate two quantitative indicators that measure a technology's 'emergingness' over time. Besides not focusing on pharmaceutical patents, they made no use of patent texts at all, unlike our proposed method. Another notable example is offered by Balsmeier and colleagues \cite{Balsmeier2018} who made use of a NLP model to visualise co-inventor networks and patenting trends in the US during the 1976-2016 period. This was achieved through using NLP to analyse the names of investors, patent holders and their locations, not too dissimilar a method from that used in the commercial AI tools for patent searches that have become available recently. Once again, no engagement occurs with the claims and content of the patents analysed, as our model does.” (pg. 6 of change-tracked file)
Comment 2. A brief discussion should be provided on the versatility of the developed AI tool and its potential to be used for patents outside of the pharmaceutical/drug space in the future.
Answer: We added the following text to discuss the versatility of the proposed methodology:  “While we focused on chemical drug patents, the method we develop can be used to identify any patent group not covered by specific IPC classes. As explained in detail, building one or more training databases with patents previously associated with the target group is a crucial step in training the algorithm. This can then be applied to the entire patent database to generalise the classification ‘learned' during training. Enabling further uses is one reason why we have explained our steps in detail.” (pg. 24 of change-tracked file)
Comment 3. Any future advancement of the developed AI tool that could improve its functionality should also be highlighted. 
Answer: Addressed in the answer above.
